# Achene Morphology and Anatomy of *Clematis* L. (Ranunculaceae) in Korea and Its Taxonomic Implications

**DOI:** 10.3390/plants9101279

**Published:** 2020-09-28

**Authors:** Balkrishna Ghimire, Beom Kyun Park, Dong Chan Son, Seung-Hwan Oh

**Affiliations:** Division of Forest Biodiversity, Korea National Arboretum, Pocheon 11186, Korea; ghimire2ab@gmail.com (B.G.); kashpbk@korea.kr (B.K.P.); sdclym@korea.kr (D.C.S.)

**Keywords:** Achene characters, *Clematis*, Ranunculaceae, taxonomic relationship

## Abstract

*Clematis*, a widely distributed genus in Ranunculaceae, is one of the most difficult groups of taxa in the family from a taxonomic point of view. A comprehensive study on achene morphology and the anatomy of 19 taxa of *Clematis* from Korea was carried out using scanning electron and light microscopy to evaluate the taxonomic significance of achene characters. *Clematis* achenes are elliptical, obovate or fusiform in shape, light yellow or brown to black in color and completely or sparsely covered with hairs. The permanent style is elongated and plumose in all the studied taxa except *C. brachyura*. We found that the size, indument, permanent style, surface sculpture, shape in cross-section, and nature and thickness of the exocarp, and endocarp were valuable achene features for species delimitation and may contribute to the unraveling of the taxonomic problems in the genus *Clematis*. One-way analysis of variance (ANOVA) indicated that the quantitative achene variables among the species were highly significant (*p* < 0.001). Principal component analyses based on seven quantitative characters and UPGMA (unweighted pair-group method with arithmetic mean) analysis based on seven quantitative and 18 qualitative characters also signify the utility of achene features for taxonomic discriminations of the *Clematis* taxa within the genus. Similar to other morphological characters in the genus *Clematis*, achene morphological and anatomical characters with the limited taxonomic value alone cannot be expected to resolve the infrageneric relationships but certain achene features combined with other morphological features could be useful as an alternative means of determining the infrageneric relationships within the genus.

## 1. Introduction

*Clematis* L. is one of the largest genera in Ranunculaceae with about 280–350 species [1,2,3] most of which are woody or herbaceous vines, but a few are shrubs, subshrubs, or erect perennial herbs. The genus is widely distributed throughout the world but with considerable diversity in temperate and subtropical regions of the Northern Hemisphere, especially eastern Asia. China, which is believed to be the center of diversity of the genus, alone has 147 species, of which 93 are endemic [4,5,6]. Its remarkable climatic plasticity, showy flowers, and easy hybridization process make *Clematis* a horticulturally important genus that is widely cultivated in Europe, North America, and East Asian countries [7].

With respect to morphology-based monographs, *Clematis* has been subjected to several infrageneric classifications [1,3,4,5,8,9]. Tamura [1] divided *Clematis* into four subgenera including 16 sections some of which were subdivided into subsections and series. Grey-Wilson [4] later grouped 297 species in nine subgenera, 16 sections, and 26 subsections whereas Johnson [5] recognized 18 sections and 36 subsections covering 325 species of the genus. Based on analyses of the various morphological and palynological characters of 345 *Clematis* species, Wang and Li [3] more recently purposed a system of classification establishing four subgenera two similar to those of Tamura [1] and two new subgeneric names within the genus, which are further divided into 15 sections and numerous subsections and series. These three classification systems largely agree with one another on species separations but vary in infrageneric discrimination. In this study, we follow Johnson [5,9] for infrageneric classification and Lee [10], Chang et al. [11], the Korea National Arboretum [12], and Kim [13] for species delimitations.

According to a comprehensive classification of Ranunculaceae by Tamura [2], *Clematis* belongs to the tribe Anemoneae of the subfamily Ranunculoideae. Furthermore, within Anemoneae the genus has been considered to be closely related to two small genera *Archiclematis* (Tamura) Tamura and *Naravelia* DC., which are together grouped in the subtribe Clamatidinae [2]. Morphologically, these three genera share a similar climbing habit and persistent hairy style in their mature achenes. The only feature that distinguishes the monotypic genus *Archiclematis* within the subtribe is the alternate leaf phyllotaxy. However, the close affinity of *Archiclematis alternata* (Kitam. and Tamura) Tamura—including similar flower morphology—with the subgenus *Viorna* Gray in *Clematis* led Wang and Li [3], Grey-Wilson [4], Wang and Bartholomew [6], and Johnson [9] to include this species within *Clematis*.

Several studies considering various morphological, anatomical, palynological, and cytological characters of *Clematis* have been performed [1,2,3,4,9,14,15,16,17,18,19,20,21,22,23,24,25]. Unfortunately, none of these morphological characters seem to provide enough information to resolve the infrageneric ambiguity of this large genus. As morphological and anatomical characters are subject to varied interpretations, the accurate infrageneric classification of this large genus, especially at the sectional level remains notoriously difficult. Due to this complex morphological variation within the genus and the different characters underlined in each system, the existing classifications systems for the genus diverge from one other [1,3,4,5,8,9]. Several molecular phylogenetic studies have also been performed in recent years [26,27,28,29,30,31] mostly supporting the monophyly of *Clematis* with clear suggestions for the retention of *Archiclematis* and *Naravelia* in a separate section within *Clematis* [27,29,30]. Unfortunately, the phylogenetic hypotheses supported by the morphological data were vastly incompatible with molecular hypotheses. Lehtonen et al. [31] recently studied 132 taxa of *Clematis* to clarify the infrageneric relationships within the genus by optimizing the phenotypic and molecular data. Their results [31] do not agree with the previous subgeneric classifications of the genus due to poor support, short branch lengths and a lack of morphologically designated units. However, they obtained 12 stable and well supported clades conceptually matching the sectional divisions of Johnson [5,9].

Fruit and seed morphological characters have contributed useful phylogenetic data and are thus frequently used to discriminate the taxa in different taxonomic ranks. In particular, the surface sculpture of fruits, seeds or a combination of both provided a valuable reference for phylogenetic and/or systematic studies [32,33,34,35,36,37,38,39,40]. Different researchers have performed fruit and seed morphological studies emphasizing the taxonomic value of several Ranunuculaceae taxa [14,39,41,42,43,44,45,46,47,48,49,50,51,52] but studies pertaining to the fruit morphology and anatomy of *Clematis* are entirely absent in previous reports. Previous molecular phylogenetic studies [27,29] concluded that many morphological features in this genus traditionally considered to be useful for determining systematic relationships were highly homoplasious and not phylogenetically indicative. However, Lehtonen et al. [31] believed that the careful re-analysis of characters may facilitate a much better character coding and understanding of morphological evolution in *Clematis*.

There is a longstanding argument among plant taxonomists regarding the exact number of species and taxonomic nomenclature of the *Clematis* taxa in Korea. Nakai [53] reported 21 species and 14 varieties of *Clematis* in a synoptical sketch of Korean flora but Lee [54] later described 16 species, 11 varieties, and five forma in the genus. Before Moon et al. [55], who claimed a new record of *C. takedana* Makino in Korea, the Korea National Arboretum and The Plant Taxonomic Society of Korea [56] included 24 taxa of *Clematis* in the synonymic list of vascular plants in Korea. Despite the new record of *C. takedana* claimed by Moon et al. [55], the origin and distribution of this species are doubtful, and not all taxonomists have accepted this taxon [10,11,12,13]. In the book New Flora of Korea, Lee [8] described 18 taxa including *C. taeguensis* Y. Lee, which was first described by Lee [57], whereas Chang et al. [8] only described 12 species and three varieties in the Illustrated Encyclopedia of Fauna & Flora of Korea. *Clematis taeguensis* was not included in the Illustrated Encyclopedia of Fauna & Flora [10] and The Flora of Korea [13] although it has been recognized as an accepted species name in the World Flora Online (WFO) [58]. The Korea National Arboretum [12] recently listed 17 species and five varieties of *Clematis* in the Checklist of Vascular Plants in Korea whereas Kim [13] described 13 species and seven varieties within the genus in The Flora of Korea. After a careful review of Lee [10], Chang et al. [11], the Korea National Arboretum [12], and Kim [13] we have included 16 species and three varieties, taking *C. takedana* as an individual species.

In this study, we provide a comprehensive investigation of achene morphology and the anatomy of 19 *Clematis* taxa distributed in Korea. The primary objective of this study was to investigate the detailed structure of achene morphology and the anatomy of the included taxa and evaluate the applicability of achene features for species discrimination. The results are also discussed in relation to the infrageneric classification of the genus.

## 2. Materials and Methods

### 2.1. Specimens

More than 550 achenes from 19 taxa representing eight sections [5,9] of *Clematis* were investigated. The names of the investigated species with their infrageneric classifications and voucher numbers are listed in Table 1. The voucher specimens are deposited in the herbarium of Korea National Arboretum (KNA).

### 2.2. Light Microscopy

At least three to five achenes of each taxon were used for microtome sectioning according to the following procedure. Mature achenes were dehydrated through an ethanol series (50, 70, 80, 90, 95 and 100%). After complete dehydration, the achenes were infiltered with ethanol/Technovit combinations (3:1, 1:1, 1:3, and 100% Technovit) and then embedded in Technovit 7100 resin. The embedded materials were cut into serial sections of 4–6 µm thickness using a Leica RM2255 rotary microtome (Leica Microsystems GmbH, Wetzlar, Germany) with disposable blades, stuck onto a slide glass, and dried using an electric slide warmer for 12 h. The dried slides were stained with 0.1% Toluidine Blue ‘O’ for 60–90 s, rinsed with water and again dried with a slide warmer for at least 6 h to remove water. The stained slides were then mounted with Entellan (Merck Co., Darmstadt, Germany) and permanent slides were prepared which were examined under a Leica DM3000 LED (Leica Microsystem, Wetzlar, Germany). Photomicrographs were taken with a scientific CMOS camera. Multiple image alignment was performed using Photoshop CS for Windows 2010. A Hirox 3D microscope (Hirox, Tokyo, Japan) was used to measure the quantitative features of the pericarp, and endocarp and the diameter of the cross section. The mean values and standard deviations of each feature were calculated from the measurements taken from the same species but different samples.

### 2.3. Scanning Electron Microscopy

The achenes were dried and desiccated thus no special pre-treatment procedures were applied in preparation for scanning electron microscopy (SEM). Before SEM imaging the achenes were immersed in 100% ethanol and were sputter coated with gold in a KIC-IA COXEM Ion-Coater (COXEM. Co., Ltd., Daejeon, Korea). SEM imaging was carried out with a COXEM EM-30 PLUS+ scanning electron microscope (COXEM) at 20 kv, at the seed testing laboratory of the Korea National Arboretum. The scale bars in the images were added manually.

### 2.4. Morphometry and Data Analysis

Approximately 400 achenes were used for morphometric measurements. Digital images of whole achenes were taken with a Leica DFC420 C multifocal camera attached to a Leica MZ16 FA microscope (Leica Microsystems). The lengths and widths of randomly selected at least 15 achenes from each taxon were measured using Leica LAS V3.8 for Windows (Supplementary File S1). The biometric data were statistically analyzed using SPSS (IBM SPSS Statistics for Windows Version 20.0., IBM Corp., Armonk, NY, USA). For each achene variable, one-factor analysis of variance (ANOVA) was used to examine differences in the means among the included species. Principal component analysis (PCA) based on means of seven quantitative (excluding ratios) achene characters and cluster analysis based on a paired group (UPGMA) of seven quantitative and 18 qualitative characters, which are categorized and coded with binary and/or multistate character states based on the standardized log10 transformations of mean values, were also performed to understand the significance of achene features for species delimitations using the statistical program PAST ver. 4.02 [59]. The character states and their coding are provided in Supplementary File S2.

## 3. Results

**Figure 1:**
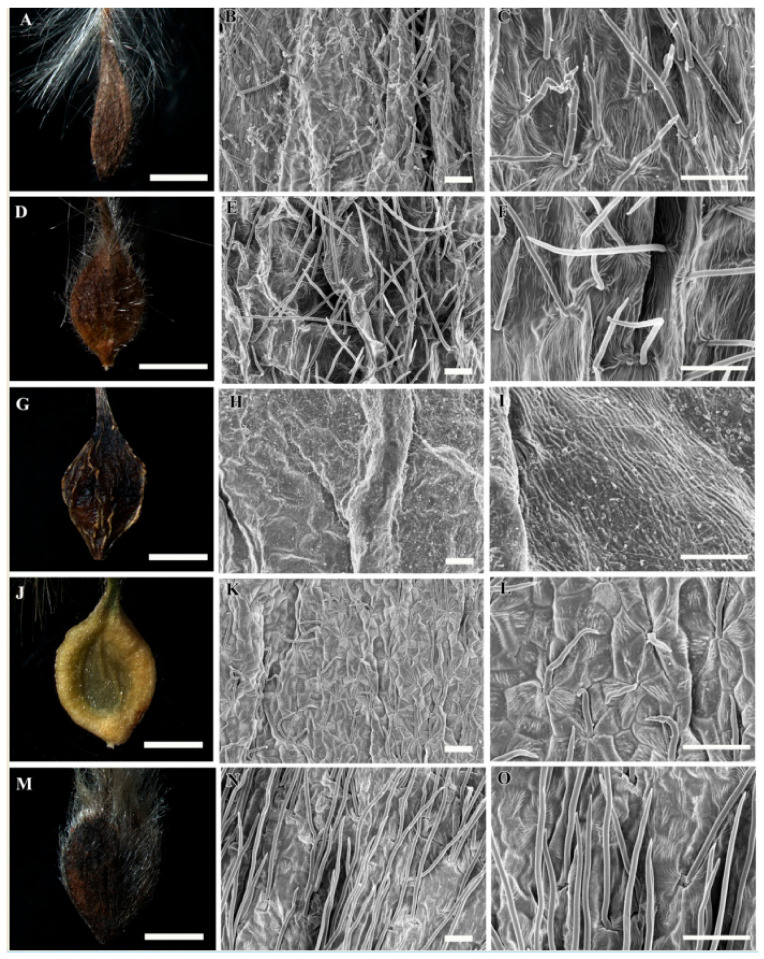
Stereomicroscopic and scanning electron microscopic micrographs of achenes of *Clematis*. (**A**–**C**) *C. apiifolia*. (**D**–**F**) *C. brevicaudata*. (**G**–**I**) *C. trichotoma*. (**J**–**L**) *C. taeguensis. (***M**–**O**) *C. hexapetala*. Scale bars: 2 mm (**A**,**D**,**G**,**J**,**M**); 100 µm (**B**,**C**,**E**,**F**,**H**,**I**,**K**,**L**,**N**,**O**).

**Figure 2:**
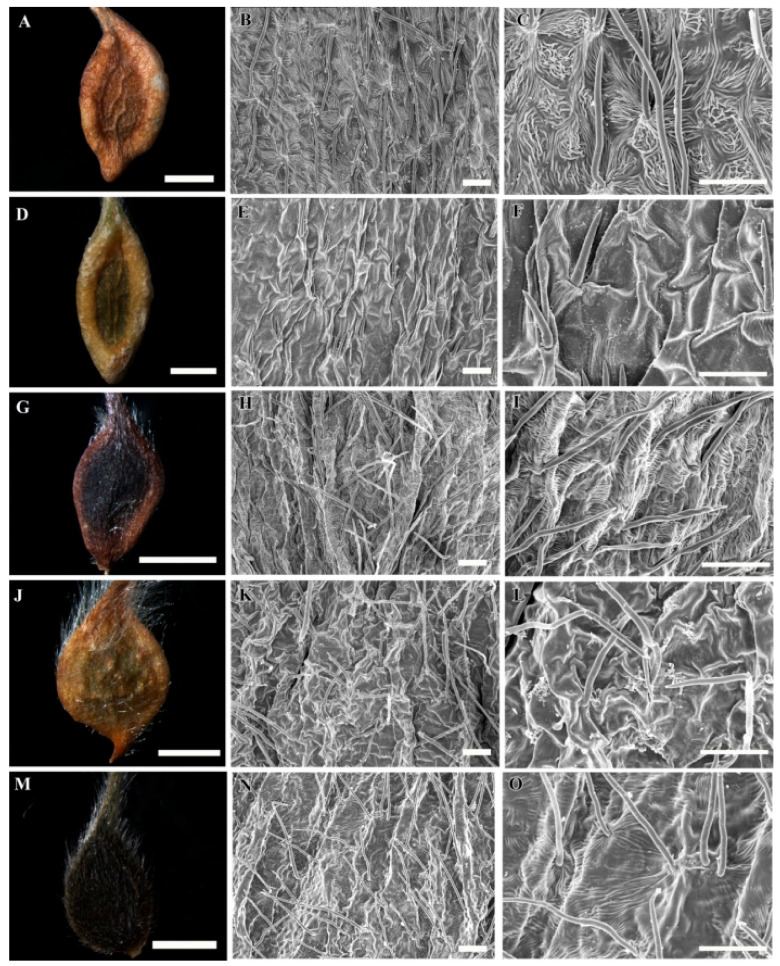
Stereomicroscopic and scanning electron microscopic micrographs of achenes of *Clematis*. (**A**–**C**) *C. terniflora*. (**D**–**F**) *C. terniflora* var. *mandshurica*. (**G**–**I**) *C. heraclefolia*. (**J**–**L**) *C. urticifolia*. (**M**–**O**) *Clematis takedana*. Scale bars: 2 mm (**A**,**D**,**G**,**J**,**M**); 100 µm (**B**,**C**,**E**,**F**,**H**,**I**,**K**,**L**,**N**,**O**).

**Figure 3:**
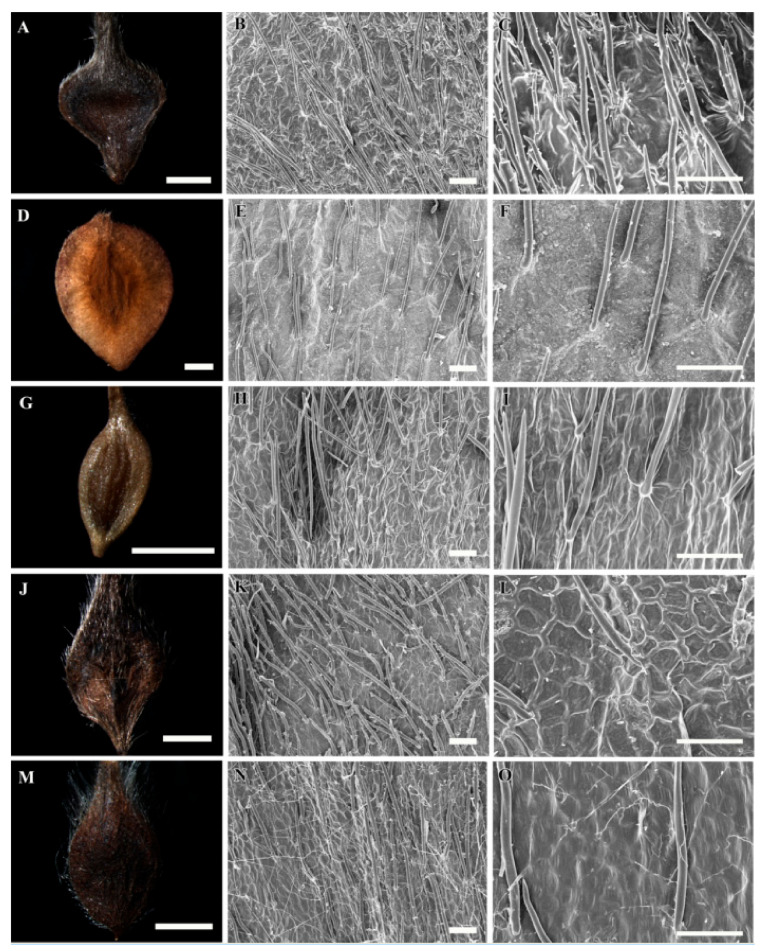
Stereomicroscopic and scanning electron microscopic micrographs of achenes of *Clematis*. (**A**–**C**) *C. patens*. (**D**–**F**) *C. brachyura*. (**G**–**I**) *C. serratifolia*. (**J**–**L**) *C. fusca* var. *fusca*. (**M**–**O**) *C. fusca* var. *flabellata*. Scale bars: 2 mm (**A**,**D**,**G**,**J**,**M**); 100 µm (**B**,**C**,**E**,**F**,**H**,**I**,**K**,**L**,**N**,**O**).

**Figure 4:**
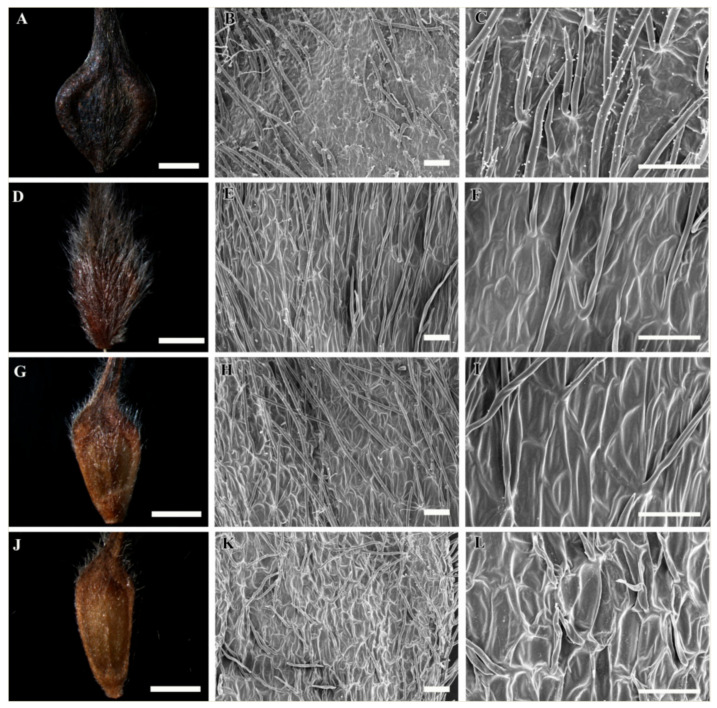
Stereomicroscopic and scanning electron microscopic micrographs of achenes of *Clematis*. (**A**–**C**) *C. fusca* var. *violacea*. (**D**–**F**) *C. calcicola*. (**G**–**I**) *C. koreana*. (**J**–**L**) *C. ochotensis*. Scale bars: 2 mm (**A**,**D**,**G**,**J**); 100 µm (**B**,**C**,**E**,**F**,**H**,**I**,**K**,**L**).

**Figure 5:**
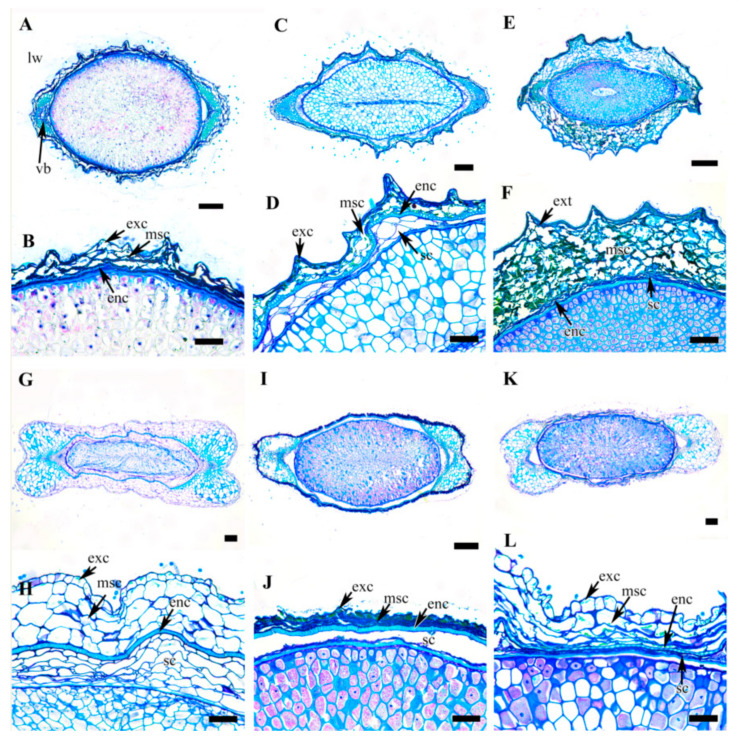
Cross section of achenes of *Clematis*. (**A**,**B**) *C. apiifolia*. (**C**,**D**) *C. brevicaudata*. (**E**,**F**) *C. trichotoma*. (**G**,**H**) *C. taeguensis*. (**I**,**J**) *C. hexapetala*. (**K**,**L**) *C. terniflora*. Abbreviations: enc, endocarp; exc, exocarp; lw, lateral wing; msc, mesocarp; sc, seed coat; vb, vascular bundle. Scale bars: 200 µm (**A**,**C**,**E**,**G**,**I**,**K**); 100 µm (**B**,**D**,**F**,**H**,**J**,**L**).

**Figure 6:**
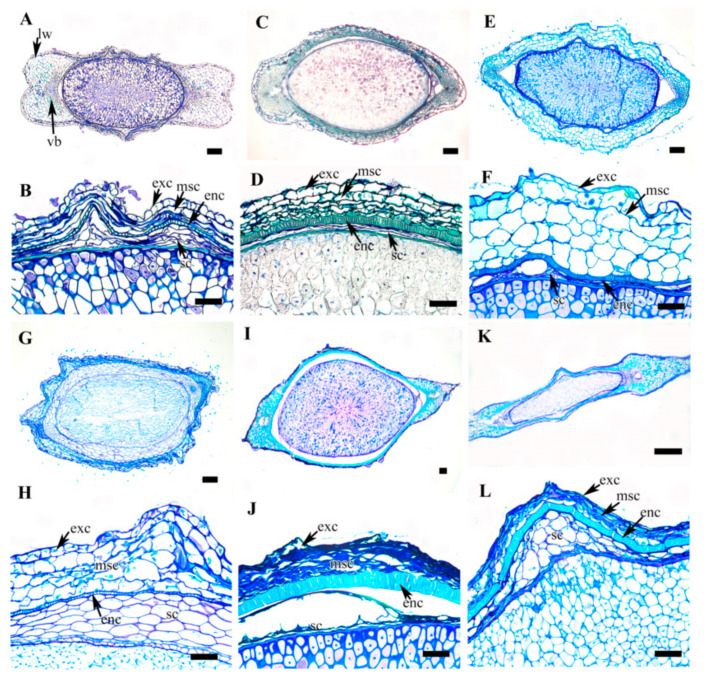
Cross section of achenes of *Clematis*. (**A**,**B**) *C. terniflora* var. *mandshurica*. (**C**,**D**) *C. heraclefolia*. (**E**,**F**) *C. urticifolia*. (**G**, **H**) *Clematis takedana*. (**I**,**J**) *C. patens*. (**K**,**L**) *C. brachyura*. Abbreviations: enc, endocarp; exc, exocarp; lw, lateral wing; msc, mesocarp; sc, seed coat; vb, vascular bundle. Scale bars: 1 mm (K), 200 µm (**A**,**C**,**E**,**G**,**I**); 100 µm (**B**,**D**,**F**,**H**,**J**,**L**).

**Figure 7:**
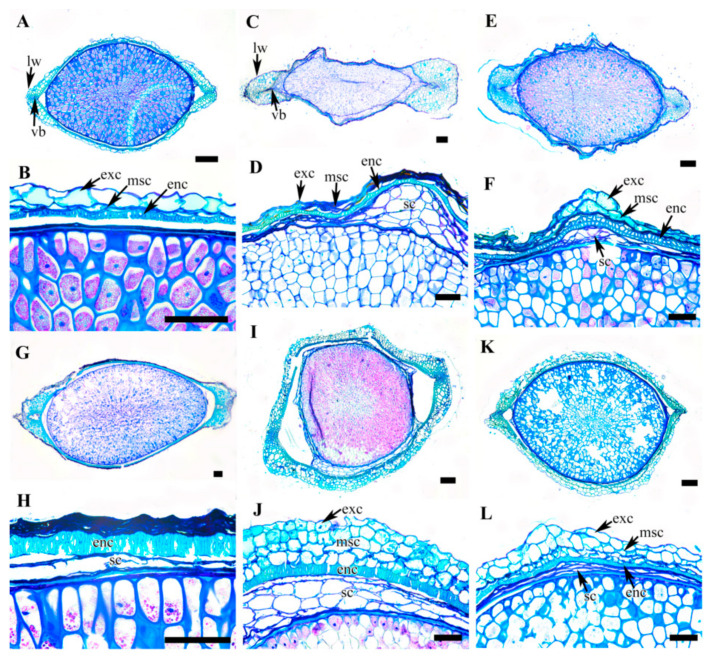
Cross section of achenes of *Clematis*. (**A**,**B**) *C. serratifolia*. (**C**,**D**) *C. fusca* var. *fusca*. (**E**,**F**) *C. fusca* var. *flabellata*. (**G**,**H**) *C. fusca* var. *violacea*. (**I**,**J**) *C. koreana*. (**K**,**L**) *C. ochotensis*. Abbreviations: enc, endocarp; exc, exocarp; lw, lateral wing; msc, mesocarp; sc, seed coat; vb, vascular bundle. Scale bars: 200 µm (**A**,**C**,**E**,**G**,**I**,**K**); 100 µm (**B**,**D**,**F**,**H**,**J**,**L**).

A selected stereomicroscopic image of a single achene, a scanning electron microscopic image of the achene surface, and light microscopic images of pericarp structure are shown in Figure 1, Figure 2, Figure 3, Figure 4, Figure 5, Figure 6, Figure 7. The morphological features of the achenes and anatomical features of the pericarp are comprehensively described below.

### 3.1. Gross Achene Morphology

The achenes of *Clematis* spp. included in this study were black, dark brown, medium brown, brown to black, light yellow and light greenish yellow in color. They were ellipsoidal, narrowly ellipsoidal, ellipsoidal to obovate, obovate, or obovate to fusiform in shape (Figure 1A,D,G,J,M, Figure 2A,D,G,J,M, Figure 3A,D,G,J,M and Figure 4A,D,G,J; Table 2). *Clematis brachyura* had the largest achene size (7.99–10.51 × 6.45–8.02 mm) followed by *C. terniflora* var. *mandshurica* (6.19–7.25 × 3.02–4.43 mm), whereas *C. bravicaudata* had the smallest (2.29–2.93 × 1.29–1.82 mm). By comparison, *C. apiifolia* had the narrowest achenes and thus higher length width ratios, whereas *C. patens* had the lowest length/width ratios (close to 1). Based on the measurements from the edges of the achenes’ bodies, the lateral wings were narrow (*C. apiifolia*, *C. bravicaudata*, *C. trochotoma*, *C. heraclefolia*, *C. serratifolia*, *C. calcicola*, *C. koreana*, and *C. ochotensis*), medium (*C. hexapetala*, *C. urticifolia*, *C. takedana*, *C. patens*, *C. fusca* var. *flabellata*, and *C. fusca* var. *violacea*), and wide (*C. taeguensis*, *C. terniflora*, *C. terniflora* var. *mandshurica*, *C. brachyura*, and *C. fusca* var. *fusca*).

### 3.2. Achene Indumentum and Style

Among the 19 taxa C. trichotoma was the only species with glabrous achene indumenta, whereas the other species were densely or sparsely pilose on the achene bodies and permanent styles (Figure 1A,D,G,J,M, Figure 2A,D,G,J,M, Figure 3A,D,G,J,M and Figure 4A,D,G,J; Table 2). Based on the achene body/permanent style length ratios, the included taxa were categorised into strongly elongated achenes (body/style ratio > 5) and elongated achenes (body/style ratio < 5). Clematis brachyura was the only exception with very short (body/style ratio < 1) and glabrous permanent styles and was thus categorised as a non elongated type. Despite the glabrous achene bodies, C. trichotoma had plumose styles.

### 3.3. Achene Surface Sculpture

The primary surface the sculptures of achenes in the studied taxa were striate-rugose: *C. apiifolia* (Figure 1B,C), *C. bravicuadata* (Figure 1E,F), *C. trichotoma* (Figure 1K,L), *C. hexapetala* (Figure 1N,O), *C. heraclefolia* (Figure 2H,I), *C. urticifolia* (Figure 2K,L), *C. takedana*. (Figure 2N,O), and *C. patens* (Figure 3B,C); striate-colliculate: *C. taguensis* (Figure 1H,I); striate-reticulate: *C. terniflora* (Figure 2B,C)*, C. terniflora* var. *mandshurica* (Figure 2E,F), *C. fusca* var. *fusca* (Figure 3K,L), *C. fusca* var. *flabellata* (Figure 3N,O), and *C. fusca* var. *violacea* (Figure 4B,C); reticulate *C. serratifolia* (Figure 3H,I), *C. calcicola* (Figure 4E,F), *C. koreana* (Figure 4H,I) and *C. ochotensis* (Figure 4K,L); and pustulate *C. brachyura* (Figure 3E,F).

### 3.4. Shape of Epidermal Cells

The surface cells were elongated (*C. hexapetala* and *C. brachyura*), elongated-rectangular (*C. taeguensis* and *C. serratifolia*), elongated-polygonal (*C. koreana* and *C. ochotensis*), rectangular-polygonal (*C. terniflora* and *C. terniflora* var. *mandshurica*), polygonal (*C. fusca* var. *fusca*, *C. fusca* var. *flabellata*, *C. violacea*, and *C. calcicola*), and irregular (*C. patens*, *C. heraclefolia*, *C. urticifolia*, and *C. takedana*). No specific cell boundaries were observed in *C. apiifolia*, *C. bravicaudata*, and *C. trichotoma*. The periclinal wall of the surface cells were dominantly concave with fine folds except in *C. taeguensis* which has convex periclinal walls with fine folds and *C. brachyura* with concave walls with tiny pustules. Likewise, the anticlinal wall was dominantly raised, smooth or folded except in *C. taeguensis* and *C. terniflora* var. *mandshurica* which had sunken and smooth anticlinal walls.

### 3.5. Achene Anatomy

The achene shapes in the cross section were mostly fusiform, elliptical, dumbbell or oval-to-elliptical in outline with narrow, medium, or wide lateral wings (Figure 5, Figure 6, Figure 7). Five taxa had elliptical (Figure 5A,C,G and Figure 6C,G), eight taxa had fusiform (Figure 5I, Figure 6E,I,K and Figure 7A,C,E,G), three taxa had dumbbell (Figure 5E,K and Figure 6A), and three taxa had oval-elliptical (Figure 7I,K; *C. calcicola* not shown) achene outlines in the cross-sections. The species with wide lateral wings had dumbbell-shaped achenes in the cross-section, except for *C. brachyura* which had large and laterally compressed achenes. The anatomical structure of the achene comprises multiple layers of thick pericarp tailed by a few layered testa of degenerating cell layer or thin-walled parenchymatous cells and a very large endosperm.

The pericarp comprised a single layered exocarp overlying a multilayered mesocarp and single layered endocarp of lignified cells. The exocarp was highly cutinized and the cells were degenerating (*C. apiifolia* and *C. fusca* var. *violacea*; Figure 5B and Figure 7H), tanniniferous (*C. bravicaudata*, *C. trichotoma*, *C. hexapetala*, *C. patens*, *C. fusca* var. *fusca*, and *C. fusca* var. *flabellata*; Figure 5D,H,J, Figure 6J and Figure 7D,F), or parenchymatous with a thick outer wall (remaining taxa including *C. calcicola*) (Figure 5F,L, Figure 6B,D,F,H,L and Figure 7B,J,L). The mesocarp was multilayered, represented by degenerating cells (*C. apiifolia*, *C. hexapetala*, *C. patens*, *C. serratifolia*, *C. fusca* var. *fusca*, *C. fusca* var. *flabellata*, and *C. fusca* var. *violacea*) or multiple layers of thin-walled parenchymatous cells (the rest of the species). The thickness of the mesocarp ranged from 2–4 layers in *C. terniflora*, *C. brachyura* and *C. ochotensis* to 8–10 layers in *C. trichotoma*. The multilayered mesocarp overlay a highly lignified single-layered or occasionally double-layered (*C. bravicaudata*, *C. brachyura*, *C. fusca* var. *flabellata*, *C. calcicola*, *C. koreana*, and *C. ochotensis*) endocarp in all the taxa. The endocarp seems to be the most consistent and vital region of the achene wall and probably serve to protect the embryo. The lignified cells may be palisade like or sclerotic (Table 2). As the pericarp takes over the protective role in the seed, the seed coat is poorly represented in mature achenes. The seed coat was represented by a few layers of degenerating cells in most of the species, while some species had several layers of thin-walled parenchymatous cells (Table 2).

The thickest pericarp was observed in *C. trichotoma* (238–525 µm) followed by *C. taeguensis* (273–420 µm), whereas the thinnest was measured in *C. serratifolia* (28.82–53.99 µm), followed by *C. hexapetala* (41.43–84.78 µm) (Table 3). The thickest endocarp was observed in *C. patens* (44.8–68.9 µm), followed by *C. fusca* var. *violacea* (29.32–39.61 µm) whereas the thinnest was measured in *C. apiifolia* (4.72–8.26 µm) followed by *C. terniflora* var. *mandshurica* (6.9–12.88 µm).

### 3.6. Statistical Analyses

**Figure 8:**
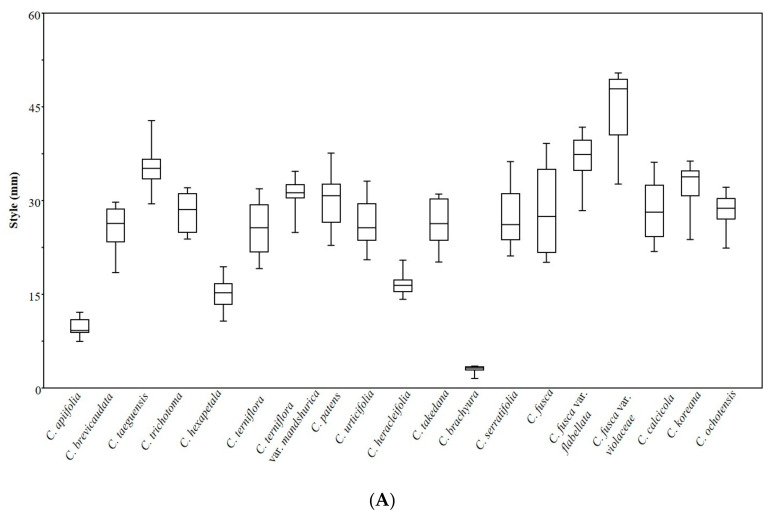

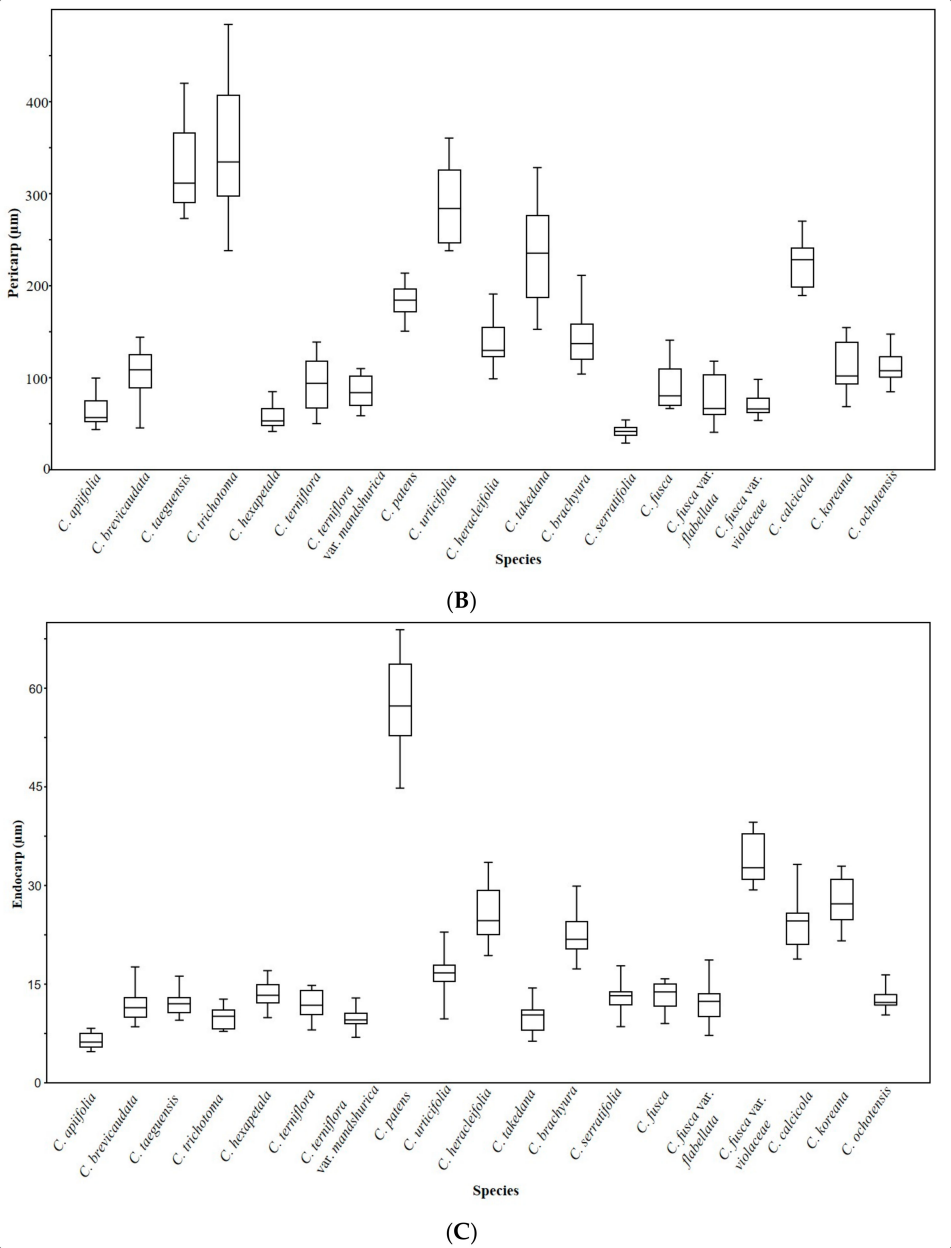
Normal boxplot showing style length, pericarp, and endocarp thickness of *Clematis* achene. (**A**) Style. (**B**) Pericarp. (**C**) Endocarp.

**Figure 9:**
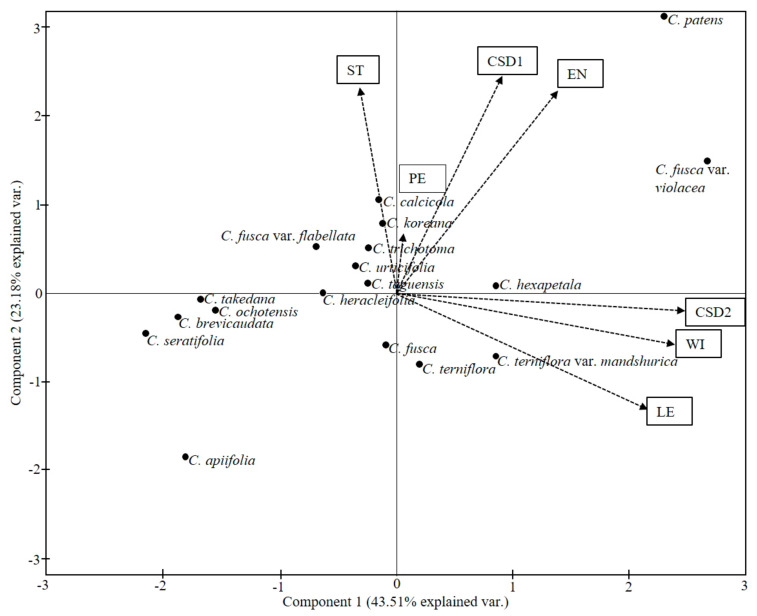
Principal component analysis (PCA) of seven quantitative achene characters of *Clematis* taxa. CSD1, cross section dimeter parallel to cotyledon; CSD2, cross section dimeter perpendicular to cotyledon; EN, thickness of endocarp; LE, length of achene; PE, thickness of pericarp; ST, length of style; WI, width.

**Figure 10:**
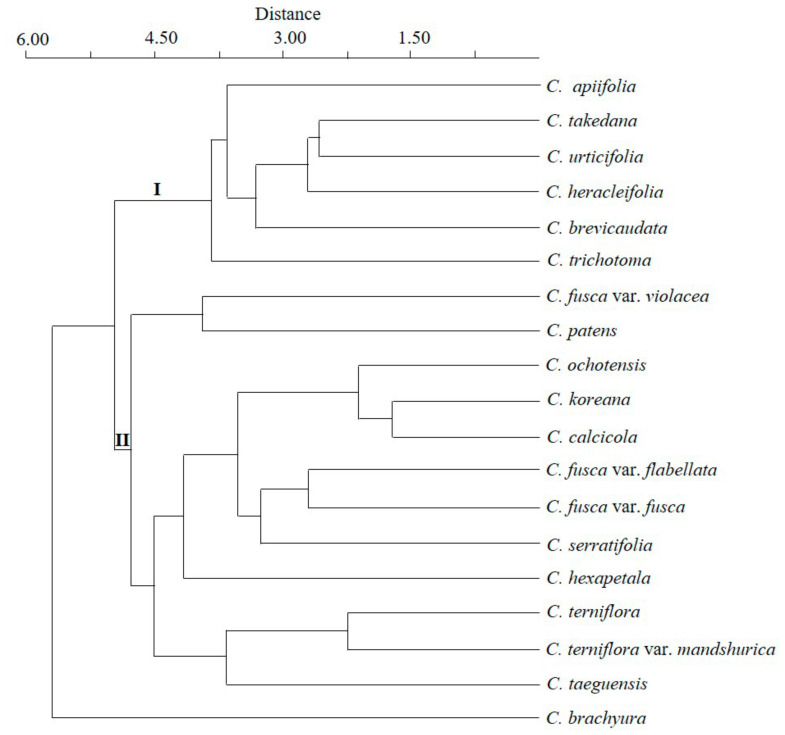
UPGMA cluster analysis based on achene characters of *Clematis* taxa.

One-factor ANOVA was performed on nine quantitative achene traits, and the differences between the species were found to be highly significant (*p* < 0.001) (Table 3). The differences in the style length and thicknesses of the pericarp and endocarp are presented in boxplots (Figure 8A–C). The similarities among the species regarding quantitative and qualitative features were revealed using PCA and cluster analysis (Figure 9). The first four components of the PCA explain 91.95% of the total variation in the analyzed data. The first axis of the first complete set explains 43.52% of the total variance and shows strong positive loadings for length, width, and cross-sectional diameter perpendicular to the embryo (LE, WE, and CSD2). The second axis explains 23.18%of the total variance and shows strong positive loadings for the style, endocarp, and cross-sectional diameter parallel to the embryo (ST, EN, and CSD1). The cluster analysis based on the paired group (UPGMA) algorithm using the Euclidean similarity index determined two large clusters uprooted with *C. brachyura* (Figure 10). The first of these represents 6 taxa whereas the second comprises the remaining 12 taxa and is further divided into at least three subclusters.

## 4. Discussion

The achenes’ morphology and anatomical features exhibited remarkable evidence for the species delimitations in *Clematis* as the studied species differ from each other by their achene morphology. The present study revealed that the achene size, pericarp and endocarp thickness, and length of permanent style were the quantitative characters that significantly differed between the species. Similarly, the achene indumentum, lateral wings, surface sculpture, achene outlines in cross-sections, and nature of the exocarp and endocarp cells were found to be helpful qualitative achene features for species identification. ANOVA of the quantitative variables revealed significant differences in achene length, width, style length, pericarp, and endocarp thickness among the taxa (*p* < 0.001) (Table 3).

Our sampling included taxa from three out of four subgenera of *Clematis* proposed by Wang and Lee [3], eight sections proposed by Johnson [5], and five clades obtained by Miikeda et al. [27], Xie et al. [28], and Lehtonen et al. [28]. There was no single described species of the subgenus *Cheiropsis* Peterm. or other sections and clades from respective classifications recorded in Korean flora until now [10,11,12,13]. As the major classification schemes based on the morphological characters of the genus do not match one another, results from recent molecular studies [27,28,31] markedly contradict such traditional infrageneric classification [1,3,4]. Since Johnson’s [5,9] classifications seem to be the most congruent with contemporary molecular phylogenetic analyses of *Clematis* we compared our results with his classification. Although the specimens sampled represented a limited range of taxa, some of the achene features and the pericarp structure represent a valuable reference for sectional delimitations by Johnson [5] within Korean *Clematis*. For instance, the achenes of three species—*C. calcicola*, *C. koreana*, and *C. ochotensis* which belong to section *Atragene*—share comparable permanent style length, reticulate primary surface sculptures, identical anticlinal walls, and endocarp cell structures. Similarly, three species of section *Tubulosae* included in this study—*C. heraclefolia*, *C. urticifolia*, and *C. takedana*—share some key achene features such as surface sculpture, surface cell outline, periclinal and anticlinal walls, and exocarp and mesocarp structure, although cross-section of *C. urticifolia* exhibit narrow lateral wings and fusiform shape instead of the medium lateral wings and elliptical shape of the other two species’ cross-sections. Correspondingly, three taxa of the section *Viorna* share equivalent pericarp thickness, striate-reticulate primary surface sculpture, polygonal surface cell outline, fusiform-shaped achenes in cross-sections, and identical mesocarp structure.

The scenario was slightly different for species of the sections *Clematis* and *Flammula*. Three species of the section *Clematis*, *C. apiifolia*, *C. brevicaudata*, and *C. trichotoma*, showed equivocally similar and dissimilar achene features. The only common feature characteristically found in these three species was the undifferentiated surface cell outline, while most of the other achene features observed in these species were shared with the species of other sections. In addition, *C. trichotoma* differed from *C. apiifolia* and *C. brevicaudata* by achene shape, color, and surface indumenta and had a comparatively thick mesocarp region. Similarly, *C. brevicaudata* had a thick layer of sclerotic endocarp and few layers of parenchymatous seed coat instead of the thin layer of lignified endocarp and degenerated seed coat found in *C. apiifolia* and *C. trichotoma*.

Four taxa of the section *Flammula*—*C. taeguensis*, *C. hexapetala*, *C. terniflora,* and *C. terniflora* var. *mandshurica*—were included and this was one of the heterogenous group in terms of achene features. Differences were observed mainly in the shape, color, surface indumentum, lateral wings, surface sculpture, periclinal wall, anticlinal wall, shape in cross-sections, thickness of the pericarp, and nature of the seed coat layers. *Clematis taeguensis* differs from the other three species by its sparsely hairy surface indumentum, strong style elongation, colliculate surface sculpture with a convex periclinal wall, and thick pericarp whereas *C. hexapetala* exhibits some unique achene features including medium-sized lateral wings, a fusiform achene outline in cross-section, a taniniferous exocarp, and few layers of degenerating mesocarp.

Although Moon et al. [55] described *C. takedana* as a newly documented species in Korea with a small population near the border of a reclaimed lake Shihwa, Hwasung-si, in Gyeonggi Province with the Korean vernacular name Ja-ju-sa-wi-jil-ppang: this species has yet to be included in The Flora of Korea [13]. *Clematis takedana* which is restricted to central and northern Honshu Island, Japan has morphological intermediacy between *C. apiifolia* (sect. *Clematis*) and *C. stans* Seibold et Zucc (sect. *Tubulosae*) and thus has been treated as a hybrid of these two species [60,61]. Wang and Bartholomew [6] suggested that this morphological intermediacy of *C. takedana* shows a transitional state between sections *Clematis* and *Tubulosae*. In the section *Tubulosae*, *C. takedana* is closely allied with *C. pinnata* Maxim. and both are placed in the same subsection *Pinnata* W. T. Wang [29,62]. Interestingly, this Chinese endemic *C. pinnata* has also been suggested to be a hybrid between *C. bravicaudata* (sect. *Clematis*) and *C. heracleifolia* (sect. *Tubulosae*) [63], indicating that the subsection *Pinnata* might be the transitional stage between sections *Clematis* and *Tubulosae*. However, recent phylogenetic study [31] denied any transitional stage and showed a very close relationship between *C. bravicaudata* and *C. heracleifolia* within a clade. Based on the achene characters we somewhat agree with the assertion that *C. takedana* is different from *C. heracleifolia* and might belong to the section *Tubulosae* but before confirming it as *C. takedana* molecular studies and investigation of the origin and distribution route of this hybrid species are necessary.

*Clematis* is taxonomically one of the most difficult genera in Ranunculaceae. There are major conflicts between morphological and molecular studies concerning the current infrageneric classifications [1,2,3,4,5,9,26,27,29,31]. Furthermore, current molecular studies have been unable to generate a robust phylogenetic framework and the infrageneric relationships remain highly unstable and poorly supported [26,27,29,31]. Cluster analyses based on seven quantitative and 18 qualitative achene features of 19 taxa have generated two large clusters connected with a basal branch representing *C. brachyura* (Figure 10).

Cluster I comprises six taxa, three from each of the sections *Tubulosae* and *Clematis*. Except for the position of individual taxa in the subclusters therein this cluster is apparently congruent with clade ‘C’ in Lehtonen et al. [31], as the section *Tubulosae* is nested in the section *Clematis*. Within the cluster *C. trichotoma* separated first followed by *C. apiifolia* and *C. brevicaudata* of section *Clematis* whereas three species of section *Tubulosae* nested in a separate subcluster. Here, *C. heracleifolia* differs from *C. urticifolia* and *C. takedana* by its fusiform- rather than elliptic-shaped achenes in cross-sections and relatively thick pericarp and endocarp. On the other hand, *C. takedana* differs from *C. heracleifolia* and *C. urticifolia* by the presence of a thick parenchymatous seed coat, with about 5–7 layers. Morphologically speaking, *C. takedana* is a woody vine whereas the other two species are perennial herbs [13,55]. Concerning the section *Clematis*, *C. trichotoma* differs from *C. brevicaudata* and *C. apiifolia* by its achene shape, color, glabrous indumentum, thick pericarp, and anticlinal walls. These three species are woody vines with 5-foliate (*C. trichotoma*), uni-ternate (*C. apiifolia*), and bi-ternate (*C. brevicaudata*) leaves [10,13]. This individual distribution of the taxa from the section *Clematis* in Cluster I indicated that achene features can be more useful for delimiting species than understanding infrageneric relationships and thus not indicative of infrageneric classification.

Cluster II comprises a morphologically heterogeneous group of taxa representing five sections included in our sampling. The most unusual grouping found in this cluster was the separation of *C. hexapetala* from other *Flammula* and rooted in the subcluster comprising the taxa of the section *Atragene*, *Viorna*, and *C. serratifolia* (section *Meclatis*). Notably, the grouping between the taxa of *Viorna* and *C. serratifolia* with *C. hexapetala* seems arbitrary here because—except for comparable pericarp thickness, shape in cross-section, and degenerated mesocarp, which are frequently evolved in other taxa as well—there is no single significant achene feature common within the group of these taxa. Regarding the three taxa of the section *Viorna*, the achenes of *C. fusca* var. *violacea*, which makes the separate subcluster with *C. patens*, are comparatively large, with thick and palisade like endocarp cells, low pericarp/endocarp ratios, and a degenerated exocarp. On the other hand, the achenes of *C. fusca* var. *flabellata* comprised an occasional bi-layered endocarp although few transversely divided endocarpic cells were also seen in *C. fusca* var. *fusca* and *C. fusca* var. *violacea*. It is worth mentioning here that *C. fusca* var. *flabellata* is an erect herb with uni-ternate leaves whereas *C. fusca* var. *fusca* and *C. fusca* var. *violacea* are woody vines with pinnately foliate leaves. Interestingly, in the Checklist of Vascular Plants in Korea [12], it is given a species name (*Clematis flabellata* Nakai); however, we cannot confirm the separate species status for *C. fusca* var. *flabellata* based on the achene morphology and anatomical evidence.

Concerning the overall similarity of *C. serratifolia* with the section *Atragene*, the group of these taxa shares a combination of achene features such as narrow lateral wings, reticulate surface sculpture, and degenerated exocarp. *Clematis serratifolia* belongs to ‘Clade I’ in Xie et al. [29] and ‘Clade I’ in Lehtonen et al. [31], whereas the section *Atragene* belongs to ‘Clades II’ in Xie et al. [29] and ‘Clade H’ in Lehtonen et al.’s [31] classifications. Equally, *C. serratifolia* differs from the species of *Atragene* by its thin pericarp, degenerated mesocarp, and palisade-like single-layered endocarp. We believe that variation in the endocarp structure is the key in *Clematis* and can be used for taxonomic discrimination, as already proved useful in other Ranunculaceae [2,8,46,51,52]. Beyond all this, *C. brachyura* remains at the bottom of the UPGMA phenogram and even out of the frame in the PCA plot based on the seven quantitative characters (Figure 9). According to Xie et al. [29], *C. brachyura* shares a clade with section *Flammula.* Previously, Tamura [1,2] and Wang and Lee [3] also suggested a close relationship between these two groups. The achenes of this morphologically isolated species are large, with sparsely distributed hairs, wide lateral wings, a lack of elongated style, and pustulate secondary surface sculpture. In terms of general morphology, *C. brachyura* shares its erect herbaceous nature, identical inflorescence, and floral structure with *C. terniflora* and *C. hexapetala* of the section *Flammula* but differs by its short style and broadly winged achenes; it is placed under the monotypic section *Pterocarpa* [2,3,4,11].

## 5. Conclusions

In this study, achene indumentum, surface sculpture, and pericarp and endocarp structure proved to be the most useful achene features for characterizing *Clematis* taxa. The results also indicated that taxa of section *Atragene* and *Tubulosae* displayed similarity in certain key achene features; however, the number of taxa sampled was very low for these sections, and thus any interpretation made on this basis is arbitrary. Further studies considering as many taxa as possible from different sections will certainly be helpful for resolving the taxonomy of the genus. The understanding from this study is that achene morphology alone as a single source of characters, cannot be expected to elucidate the problematic infrageneric relationships but a thorough analysis of current and other morphological, as well as molecular data, will be helpful to accomplish this. Our results, however, demonstrated that achene features can contribute valuable information and could be used as descriptive and/or diagnostic characters of the *Clematis* species.

## Figures and Tables

**Table 1:** Name of taxa with voucher number and collection information.

Taxon	Locality	Voucher No.
*C. apiifolia* DC.	Mt. Sinbul, Icheon-ri, Sangbuk-myeon, Ulju-gun, Ulsan, Korea	Sinbulsan-190911-001
*C. brevicaudata* DC.	Unchi-ri, Sindong-eup, Jeongseon-gun, Gangwon-do, Korea	Unchiri-191007-001
*C. trichotoma* Nakai	Mt. Sinbul, Icheon-ri, Sangbuk-myeon, Ulju-gun, Ulsan, Korea	Sinbulsan-190911-001
*C. taeguensis* Y. Lee	Gyuram-ri, Jeongseon-eup, Jeongseon-gun, Gangwon-do, Korea	Gyuramri-190818-001
*C. hexapetala* Pall.	Ho-ri, Palbong-myeon, Seosan-si, Chungcheongnam-do, Korea	Hori-190809-001
*C. terniflora* DC.	Jukpo-ri, Dolsan-eup, Yeosu-si, Jeollanam-do, Korea	Dolsando-191004-002
*C. terniflora* var. *mandshurica* (Rupr.) Ohwi	Namhansanseong Fortress, Sanseong-ri, Namhansanseong-myeon, Gwangju-si, Gyeonggi-do, Korea	Namhansanseong-190809-001
*C. heracleifolia* DC.	Sihwa Lake, Munho-ri, Namyang-eup, Hwaseong-si, Gyeonggi-do, Korea	Sihwaho-190921-016
*C.urticifolia* Nakai ex Kitag.	Mt. Gariwang, Sugam-ri, Bukpyeong-myeon, Jeongseon-gun, Gangwon-do, Korea	Gariwangsan-191007-001
*C. takedana* Makino	Sihwa Lake, Munho-ri, Namyang-eup, Hwaseong-si, Gyeonggi-do, Korea	Sihwaho-190921-001
*C. patens* C.Morren & Dence.	Mt. Johang, Samsong-ri, Cheongcheon-myeon, Goesan-gun, Chungcheongbuk-do, Korea	Johangsan-170831-049
*C. brachyura* Maxim.	Seondol, Bangjeol-ri, Yeongwol-eup, Yeongwol-gun, Gangwon-do, Korea	Seondol-190719-001
*C. serratifolia* Rehder	Gasong-ri, Dosan-myeon, Andong-si, Gyeongsangbuk-do, Korea	Gasongri-191007-001
*C. fusca* var. *fusca* Turcz.	Mt. Cheongtae, Sapgyo-ri, Dunnae-myeon, Hoengseong-gun, Gangwon-do, Korea	Cheongtaesan-190819-001
*C. fusca* var. *flabellata* (Nakai) J. S. Kim	Eundae-bong, Gohan-ri, Gohan-eup, Jeongseon-gun, Gangwon-do, Korea	Eundaebong-190818-001
*C. fusca* var. *violacea* Maxim.	Mt. Baekhwa, Mawon-ri, Mungyeong-eup, Mungyeong-si, Gyeongsangbuk-do, Korea	Baekhwasan-150707-007
*C. calcicola* J. S. Kim	Mt. Deokhang, Daei-ri, Singi-myeon, Samcheok-si, Gangwon-do, Korea	Deokhangsan-190818-001
*C. koreana* Kom.	Mt. Hambaek, Gohan-ri, Gohan-eup, Jeongseon-gun, Gangwon-do, Korea	Hambaeksan-190818-001
*C. ochotensis* (Pall.) Poiret	Mt. Gariwang, Sugam-ri, Bukpyeong-myeon, Jeongseon-gun, Gangwon-do, Korea	Gariwangsan-190819-007

**Table 2:** Morphological and anatomical features of achene of *Clematis.*

Taxon	Shape	Color	Achene Indumentum	Lateral Wings	Style Elongation	Style	Surface Sculpture	Surface Cells Outline	Periclinal Wall	Anticlinal Wall
*C. apiifolia*	Narrow-elliptical	Brown	Completely hairy, hairs short on the achene body and long on the style	Narrow	Elongated	Plumose	Striate-rugose	Not differentiated	Concave with fine folds	Raised, folded
*C. brevicaudata*	Elliptic	Brown	Completely hairy, hairs short on the achene body and long on the style	Narrow	Strongly elongated	Plumose	Striate-rugose	Not differentiated	Concave with fine folds	Raised, folded
*C. trichotoma*	Obovate to fusiform	Black	Glabrous	Narrow	Strongly elongated	Plumose	Striate-rugose	Not differentiated	Concave with fine folds	Slightly raised
*C. taeguensis*	Elliptical to obovate	Light yellow	Sparsely hairy, fine hair on the body and long on the style	Wide	Strongly elongated	Plumose	Striate-colliculate	Elongated, rectangular	Convex with fine folds	Sunken, smooth
*C. hexapetala*	Obovate	Dark brown to black	Completely hairy	Medium	Elongated	Plumose	Striate-rugose	Elongated	Concave with fine folds	Raised, folded
*C. terniflora*	Elliptic to Obovate	Brown	Sparsely hairy, fine hair on the body and long on the style	Wide	Elongated	Plumose	Striate-reticulate	Rectangular, polygonal	Concave with fine folds	Raised, smooth
*C. terniflora* var. *mandshurica*	Elliptic	Light yellow	Sparsely hairy, fine hair on the body and long on the style	Wide	Elongated	Plumose	Striate-reticulate	Rectangular, polygonal	Concave with fine folds	Sunken, smooth
*C. heracleifolia*	Obovate	Dark brown	Completely hairy	Narrow	Strongly elongated	Plumose	Striate-rugose	Irregular	Concave with fine folds	Raised, folded
*C. urticifolia*	Elliptic to Obovate	Light greenish yellow	Completely hairy	Medium	Strongly elongated	Plumose	Striate-rugose	Irregular	Concave with fine folds	Raised, folded
*C. takedana*	Obovate	Dark brown to black	Completely hairy	Medium	Strongly elongated	Plumose	Striate-rugose	Irregular	Concave with fine folds	Raised, folded
*C. patens*	Obovate	Dark brown to black	Completely hairy, hairs short on the achene body and long on the style	Medium	Strongly elongated	Plumose	Striate-rugose	Irregular	Concave with fine folds	Raised, folded
*C. brachyura*	Obovate	Brown, black	Sparsely hairy	Wide	Not elongated	Glabrous	Rugose, Pustulate	Elongated	Concave pustulate	Raised, pustulate
*C. serratifolia*	Obovate	Medium brown	Sparsely hairy, fine hair on the body and long on the style	Narrow	Strongly elongated	Plumose	Reticulate	Elongated, rectangular	Concave with fine folds	raised, smooth
*C. fusca* var. *fusca*	Obovate	Dark brown to black	Completely hairy	Wide	Strongly elongated	Plumose	Striate-reticulate	Polygonal	Concave with fine folds	Raised smooth or fine folds
*C. fusca* var. *flabellata*	Elliptic to Obovate	Brown	Completely hairy	Medium	Strongly elongated	Plumose	Striate-reticulate	Polygonal	Concave with fine folds	Slightly raised, smooth
*C. fusca* var. *violacea*	Obovate	Black	Completely hairy	Medium	Strongly elongated	Plumose	Striate-reticulate	Polygonal	Concave with fine folds	Raised smooth or fine folds
*C. calcicola*	Narrow-elliptical	Dark brown	Completely hairy	Narrow	Strongly elongated	Plumose	Reticulate	Polygonal	Concave with fine folds	Raised smooth or fine folds
*C. koreana*	Obovate	Medium brown	Completely hairy	Narrow	Strongly elongated	Plumose	Reticulate	Elongated, polygonal	Concave with fine folds	Raised smooth or fine folds
*C. ochotensis*	Obovate	Brown	Sparsely hairy, fine hairs on the body and long on the style	Narrow	Strongly elongated	Plumose	Reticulate	Elongated, polygonal	Concave with fine folds	Raised smooth or fine folds
Taxon		Outline in CS	Exocarp		Mesocarp		Endocarp		Seed Coat	
*C. apiifolia*		Elliptic	Degenerating cells		Few layers of degenerating cells		Thin, single layer of lignified cells		Degenerated	
*C. brevicaudata*		Elliptic	Cutinized single tanniniferous layer		3–4 layers of parenchyma layer with wavy wall		Thick, single layer (rarely double layer) sclerid cells		Few layers of parenchyma cells	
*C. trichotoma*		Elliptic	Cutinized single tanniniferous layer		8–10 layers of thin walled parenchyma cells with wavy wall		Thin, single layer of lignified cells		1–2 layers of degenerating cells	
*C. taeguensis*		Dumbbell	Cutinized single layer parenchyma cells with thick outer wall		4–6 layers of thin walled parenchyma cells with wavy wall		Thick, single layer of sclerid cells		5–7 layers of parenchyma cells	
*C. hexapetala*		Fusiform	Cutinized single tanniniferous layer		Few layers of degenerating cells		Thick, single layer of sclerid cells		Few layers of degenerating cells	
*C. terniflora*		Dumbbell	Cutinized single layer parenchyma cells with thick outer wall		2–4 layers of thin walled parenchyma cells with wavy wall		Single layer of sclerid cells		Few layers of degenerating cells	
*C. terniflora* var. *mandshurica*		Dumbbell	Cutinized single layer parenchyma cells with thick outer wall		4–6 layers of thin walled parenchyma cells with wavy wall		Thick, single layer of sclerid cells		Few layers of parenchyma cells	
*C. heracleifolia*		Fusiform	Cutinized single layer parenchyma cells with thick outer wall		4–6 layers of thin walled parenchyma cells with wavy wall		Thick, single layer of lignified palisade cells		Few layers of degenerating cells	
*C. urticifolia*		Elliptic	Cutinized single layer parenchyma cells with thick outer wall		4–6 layers of thin walled parenchyma cells with wavy wall		Single layer of sclerid cells		Few layers of degenerating cells	
*C. takedana*		Elliptic	Cutinized single layer parenchyma cells with thick outer wall		6–8 layers of thin walled parenchyma cells with wavy wall		Thin, single layer of lignified cells		5–7 layers of parenchyma cells	
*C. patens*		Fusiform	Cutinized single tanniniferous layer		Few layers of degenerating cells		Thick, single layer of lignified palisade cells		Few layers of parenchyma cells	
*C. brachyura*		Narrowly fusiform	Cutinized single layer parenchyma cells with thick outer wall		2–4 layers of thin walled parenchyma cells with wavy wall		Thick, single layer (rarely double layer) sclerid cells		3–5 layers of parenchyma cells	
*C. serratifolia*		Fusiform	Cutinized single layer parenchyma cells with thick outer wall		Few layers of degenerating cells		Thick, single layer of lignified palisade cells		Few layers of degenerating cells	
*C. fusca* var. *fusca*		Fusiform	Cutinized single taniniferous layer		Few layers of degenerating cells		Single layer of sclerid cells		5–7 layers of parenchyma cells	
*C. fusca* var. *flabellata*		Fusiform	Cutinized single taniniferous layer		Few layers of degenerating cells		Thick, single layer (rarely double layer) sclerid cells		Few layers of degenerating cells	
*C. fusca* var. *violacea*		Fusiform	Degenerated		Few layers of degenerating cells		Thick, single layer of lignified palisade cells		Few layers of degenerating cells	
*C. calcicola*		Oval, elliptical	Cutinized single layer parenchyma cells with thick outer wall		6–8 layers of thin walled parenchyma cells with wavy wall		Thick, single layer (rarely double layer) sclerid cells		Few layers of degenerating cells	
*C. koreana*		Oval, elliptical	Cutinized single layer parenchyma cells with thick outer wall		6–8 layers of thin walled parenchyma cells with wavy wall		Thick, single layer (rarely double layer) sclerid cells		3–5 layers of parenchyma cells	
*C. ochotensis*		Oval, elliptical	Cutinized single layer parenchyma cells with thick outer wall		2–3 layers of thin walled parenchyma cells with wavy wall		Thin, single layer (rarely double layer) sclerid cells		Few layers of degenerating cells	

**Table 3:** One-factor ANOVA for the achene characters and their measurements in *Clematis* taxa (mean and standard deviation). L/W = length and width ratio, P/E = pericarp and endocarp ratio, S/B = style and achene body ratio, CSD1 = diameter parallel to cotyledon, CSD2 = diameter perpendicular to cotyledon, D1/D2 = CSD1 and CSD2 ratio.

Taxon	Length (mm)	Width (mm)	L/W	Pericarp (µm)	Endocarp (µm)	P/E	Style (mm)	S/B	CSD1 (mm)	CSD2 (mm)	CSD1/CSD2
*C. apiifolia*	3.95 ± 0.4	1.32 ± 0.2	3.01 ± 0.21	63.51 ± 15.58	6.32 ± 1.06	10 ± 1.36	9.55 ± 1.29	2.44 ± 0.38	1.66 ± 0.04	1.08 ± 0.07	1.54 ± 0.08
*C. brevicaudata*	2.53 ± 0.19	1.62 ± 0.18	1.59 ± 0.21	104.89 ± 26.35	12.03 ± 3	8.99 ± 2.56	25.74 ± 3.24	10.17 ± 1.17	2.07 ± 0.05	1.15 ± 0.09	1.8 ± 0.15
*C. trichotoma*	4 ± 0.27	2.67 ± 0.24	1.5 ± 0.05	343.62 ± 68.40	9.97 ± 1.52	35.94 ± 6.86	27.74 ± 3.04	6.95 ± 0.83	3.06 ± 0.28	2.17 ± 0.39	1.43 ± 0.16
*C. taeguensis*	4.3 ± 0.47	3.3 ± 0.48	1.34 ± 0.21	327.13 ± 46.45	12.06 ± 1.64	27.19 ± 2.17	35.35 ± 3.48	8.25 ± 1.2	3.28 ± 0.08	1.15 ± 0.1	2.87 ± 0.24
*C. hexapetala*	4.73 ± 0.4	2.88 ± 0.2	1.64 ± 0.04	56.52 ± 11.77	13.47 ± 1.93	4.17 ± 0.35	15.01 ± 2.33	3.21 ± 0.63	2.93 ± 0.21	1.38 ± 0.19	2.13 ± 0.14
*C. terniflora*	5.44 ± 0.43	3.12 ± 0.3	1.75 ± 0.06	94.85 ± 29.49	11.84 ± 2.05	7.83 ± 1.24	25.97 ± 4.08	4.76 ± 0.74	3.44 ± 0.3	1.33 ± 0.14	2.6 ± 0.14
*C. terniflora* var. *mandshurica*	6.75 ± 0.39	3.5 ± 0.4	1.94 ± 0.13	84.89 ± 16.41	9.84 ± 1.43	8.72 ± 1.65	31.66 ± 3.24	4.71 ± 0.55	3.73 ± 0.21	1.47 ± 0.12	2.55 ± 0.21
*C. heracleifolia*	3.21 ± 0.36	2.01 ± 0.25	1.6 ± 0.0.08	136.06 ± 25.36	25.61 ± 4.41	5.3 ± 0.17	17.07 ± 2.55	5.4 ± 1.21	3.01 ± 0.48	1.42 ± 0.12	2.21 ± 0.18
*C. urticifolia*	3.83 ± 0.25	2.9 ± 0.27	1.33 ± 0.09	285.8 ± 41.04	16.35 ± 3.12	17.24 ± 4.21	26.55 ± 3.8	6.97 ± 1.25	2.89 ± 0.37	1.49 ± 0.09	1.94 ± 0.33
*C. takedana*	2.88 ± 0.38	1.86 ± 0.22	1.56 ± 0.21	232.31 ± 52.36	9.94 ± 2.27	23.92 ± 5.1	26.23 ± 3.7	9.3 ± 1.64	2.08 ± 0.08	1.27 ± 0.16	1.66 ± 0.26
*C. patens*	4.04 ± 0.3	3.68 ± 0.51	1.12 ± 0.16	183.29 ± 18.69	57.69 ± 7.41	3.33 ± 0.83	30.21 ± 5.17	7.51 ± 1.41	4.95 ± 0.2	2.45 ± 0.13	2.03 ± 0.18
*C. brachyura*	9.12 ± 0.88	7.3 ± 0.46	1.25 ± 0.10	142.19 ± 28.08	22.44 ± 3.39	6.37 ± 1.04	2.99 ± 0.55	0.33 ± 0.07	7.1 ± 0.14	1.08 ± 0.11	6.65 ± 0.6
*C. serratifolia*	2.86 ± 0.17	1.42 ± 0.13	2.02 ± 0.11	41.44 ± 6.93	12.83 ± 2.09	3.23 ± 0.14	27.16 ± 4.52	9.51 ± 1.62	1.61 ± 0.04	1.04 ± 0.1	1.6 ± 0.12
*C. fusca* var. *fusca*	4.64 ± 0.41	2.99 ± 0.28	1.52 ± 0.07	89.63 ± 25.9	13.29 ± 1.93	6.9 ± 2.26	28.61 ± 6.65	6.25 ± 1.71	3.58 ± 0.17	1.21 ± 0.08	2.96 ± 0.2
*C. fusca* var. *flabellata*	3.77 ± 0.3	2.49 ± 0.23	1.14 ± 0.06	77.92 ± 25.31	12.51 ± 2.71	6.3 ± 0.95	35.71 ± 4.9	9.55 ± 1.72	2.86 ± 0.11	1.65 ± 0.08	1.73 ± 0.02
*C. fusca* var. *violacea*	6.1 ± 0.7	5.34 ± 0.51	1.18 ± 0.38	70.3 ± 12.45	34.1 ± 3.54	2.05 ± 0.16	44.74 ± 5.81	7.37 ± 0.9	5.16 ± 0.15	1.92 ± 0.32	2.73 ± 0.34
*C. calcicola*	4.44 ± 0.48	2.07 ± 0.25	2.18 ± 0.38	222.95 ± 25.82	24.27 ± 4.22	9.35 ± 2.25	28.48 ± 4.29	6.48 ± 1.23	2.86 ± 0.25	1.85 ± 0.3	1.59 ± 0.33
*C. koreana*	4.77 ± 0.35	2.55 ± 0.36	1.88 ± 0.12	111.82 ± 26.78	27.66 ± 3.51	4.11 ± 0.46	31.85 ± 4.95	6.7 ± 1.14	2.36 ± 0.07	1.66 ± 0.08	1.42 ± 0.04
*C. ochotensis*	3.76 ± 0.25	1.64 ± 0.26	2.35 ± 0.39	111.83 ± 16.44	12.57 ± 1.5	8.96 ± 1.42	28.56 ± 2.45	7.64 ± 0.89	1.81 ± 0.19	1.29 ± 0.15	1.42 ± 0.3
ANOVA	F = 206.22, *p* < 0.001	F = 298.9, *p* < 0.001	F = 101.98, *p* < 0.001	F = 121.01, *p* < 0.001	F = 182.24, *p* < 0.001	F = 190.03, *p* < 0.001	F = 83.7, *p* < 0.001	F = 72.68, *p* < 0.001	F = 210.17, *p* < 0.001	F = 26.89, *p* < 0.001	F = 114.58, *p* < 0.001

## Supplementary Materials

The following are available online at https://www.mdpi.com/2223-7747/9/10/1279/s1, File S1: Quantitative measurement of achene features of *Clematis* species, File S2: Character and character states of achenes of *Clematis*.
