# Supplementary material for: Achene Morphology and Anatomy of Clematis L. (Ranunculaceae) in Korea and Its Taxonomic Implications
Source: Plants (Basel). 2020 Sep 28;9(10):1279. doi: 10.3390/plants9101279 (PMC7650620; doi:10.3390/plants9101279)
Supplement: Supplementary file 1 [file plants-09-01279-s001.zip › supplementary/S2_Supplementary_Characters coding_new.doc]

**S2: Character and character states of achenes of *Clematis*.**

**Quantitative traits of achene:**

1. Achene length (L): <3 mm, 1; 3-5 mm, 2; >5 mm, 3.
2. Achene width (W): <3 mm, 1; 3-5 mm, 2; >5 mm, 3.
3. Pericarp thickness (P): <100 µm, 100-200 µm, 2; >200 µm, 3.
4. Endocarp thickness (E): <15 µm, 1; 15-40<20 µm, 2; >40 µm, 3.
5. Style length: <5 mm, 5-20 mm, 2; >20 mm, 3.
6. Cross section diameter (CSD) parallel to embryo: 1-2 mm, 1; 2-4 mm, 2; >4 mm, 3.
7. CSD perpendicular to embryo: 1-1.5 mm, 1; 1.5-2 mm, 2; >2 mm, 3.

**Qualitative features of achene:**

8. Achene shape: elliptical, 1; obovate, 2
9. Achene color: Brown, 1; other, 2.
10. Achene indumentum: Completely hairy, 1; Sparsely hairy, 2; Glabrous, 3
11. Lateral wings: Narrow, 1; medium, 2; wide, 3.
12. Style elongation: not elongated, 1; elongated, 2; strongly elongated, 3.
13. Style indumentum: Glabrous, 1; Plumose, 2.
14. Surface sculpture: Striate-rugose, 1; Striate-colliculate, 2; Striate-reticulate, 3; Reticulate, 4.
15. Surface cell outline: Elongated, 1; Elongated-polygonal/Elongated-rectangular, 2; Rectangular-polygonal, 3; Irregular, 4; not differentiated, 5
16. Surface periclinal walls: Concave, 1; convex, 2.
17. Surface anticlinal walls: Raised, 1; slightly raised, 2; sunken, 3.
18. Anticlinal wall secondary ornamentation: Folded, 1; smooth, 2.
19. Achene outline in cross section: Elliptical, 1; Fusiform, 2; Dumbbell, 3.
20. Exocarp nature: Degenerated, 1; well represented, 2.
21. Exocarp cell type: Parenchymatous, 1; tanniniferous, 2.
22. Mesocarp: Degenerated, 1; well represented, 2.
23. Endocarp cell nature: Palisade, 1; sclerotic, 2.
24. Endocarp layer: single layer, 1; occasionally two layers, 2.
25. Seed coat: Degenerated, 1; well represented, 2.

| **Taxon** | **1** | **2** | **3** | **4** | **5** | **6** | **7** | **8** | **9** | **10** | **11** | **12** | **13** | **14** | **15** | **16** | **17** | **18** | **19** | **20** | **21** | **22** | **23** | **24** | **25** |
| --- | --- | --- | --- | --- | --- | --- | --- | --- | --- | --- | --- | --- | --- | --- | --- | --- | --- | --- | --- | --- | --- | --- | --- | --- | --- |
| *C. apiifolia* | 2 | 1 | 1 | 1 | 2 | 1 | 1 | 1 | 1 | 1 | 1 | 2 | 2 | 1 | 5 | 1 | 1 | 1 | 1 | 1 | ? | 1 | 1 | 1 | 1 |
| *C. brevicaudata* | 1 | 1 | 2 | 1 | 3 | 2 | 1 | 1 | 1 | 1 | 1 | 3 | 2 | 1 | 5 | 1 | 1 | 1 | 1 | 2 | 2 | 2 | 2 | 2 | 2 |
| *C. trichotoma* | 2 | 1 | 3 | 1 | 3 | 2 | 2 | 2 | 2 | 3 | 1 | 3 | 2 | 1 | 5 | 1 | 2 | 2 | 1 | 1 | 2 | 2 | 1 | 1 | 2 |
| *C. taeguensis* | 2 | 2 | 3 | 1 | 3 | 2 | 1 | 1 | 2 | 2 | 3 | 3 | 2 | 2 | 2 | 2 | 3 | 2 | 3 | 1 | 1 | 2 | 2 | 1 | 2 |
| *C. hexapetala* | 2 | 1 | 1 | 1 | 2 | 2 | 2 | 2 | 1 | 1 | 2 | 2 | 2 | 1 | 1 | 1 | 1 | 1 | 2 | 1 | 2 | 1 | 2 | 1 | 1 |
| *C. terniflora* | 3 | 2 | 1 | 1 | 3 | 2 | 1 | 1,2 | 1 | 2 | 3 | 2 | 2 | 3 | 3 | 1 | 1 | 2 | 3 | 1 | 1 | 2 | 2 | 1 | 1 |
| *C. terniflora* var. *mandshurica* | 3 | 2 | 1 | 1 | 3 | 2 | 1 | 1 | 2 | 2 | 3 | 2 | 2 | 3 | 3 | 1 | 3 | 2 | 3 | 1 | 1 | 2 | 2 | 1 | 1 |
| *C. heracleifolia* | 2 | 1 | 2 | 2 | 2 | 2 | 1 | 2 | 1 | 1 | 1 | 3 | 2 | 1 | 4 | 1 | 1 | 1 | 2 | 1 | 1 | 2 | 1 | 1 | 1 |
| *C. urticifolia* | 2 | 1 | 3 | 2 | 3 | 2 | 2 | 1,2 | 2 | 1 | 2 | 3 | 2 | 1 | 4 | 1 | 1 | 1 | 1 | 1 | 1 | 2 | 2 | 1 | 1 |
| *C. takedana* | 1 | 1 | 3 | 1 | 3 | 2 | 1 | 2 | 1 | 1 | 2 | 3 | 2 | 1 | 4 | 1 | 1 | 1 | 1 | 1 | 1 | 2 | 1 | 1 | 2 |
| *C. patens* | 2 | 2 | 2 | 3 | 3 | 3 | 3 | 2 | 1 | 1 | 2 | 3 | 2 | 1 | 4 | 1 | 1 | 1 | 2 | 1 | 2 | 1 | 1 | 1 | 2 |
| *C. brachyura* | 3 | 3 | 2 | 2 | 1 | 3 | 1 | 2 | 1 | 2 | 3 | 1 | 1 | 1 | 1 | 1 | 1 | 1 | 2 | 1 | 1 | 2 | 2 | 2 | 2 |
| *C. serratifolia* | 1 | 1 | 1 | 1 | 3 | 1 | 1 | 2 | 1 | 2 | 1 | 3 | 2 | 3 | 2 | 1 | 1 | 2 | 2 | 1 | 1 | 1 | 1 | 1 | 1 |
| *C. fusca* var. *fusca* | 2 | 2 | 1 | 1 | 3 | 2 | 1 | 2 | 1 | 1 | 3 | 3 | 2 | 3 | 2 | 1 | 1 | 1,2 | 2 | 1 | 2 | 1 | 2 | 1 | 2 |
| *C. fusca* var. *flabellata* | 2 | 1 | 1 | 1 | 3 | 2 | 2 | 1,2 | 1 | 1 | 2 | 3 | 2 | 3 | 2 | 1 | 2 | 2 | 2 | 1 | 2 | 1 | 2 | 2 | 1 |
| *C. fusca* var. *violacea* | 3 | 3 | 1 | 2 | 3 | 3 | 3 | 2 | 2 | 1 | 2 | 3 | 2 | 3 | 2 | 1 | 1 | 1,2 | 2 | 2 | ? | 1 | 1 | 1 | 1 |
| *C. calcicola* | 2 | 1 | 3 | 2 | 3 | 2 | 2 | 1 | 1 | 1 | 1 | 3 | 2 | 4 | 2 | 1 | 1 | 1,2 | 1 | 1 | 1 | 2 | 2 | 2 | 1 |
| *C. koreana* | 2 | 1 | 2 | 2 | 3 | 2 | 2 | 2 | 1 | 1 | 1 | 3 | 2 | 4 | 2 | 1 | 1 | 1,2 | 1 | 1 | 1 | 2 | 2 | 2 | 2 |
| *C. ochotensis* | 2 | 1 | 2 | 1 | 3 | 1 | 1 | 2 | 1 | 1 | 1 | 3 | 2 | 4 | 2 | 1 | 1 | 1,2 | 1 | 1 | 1 | 2 | 2 | 2 | 1 |
