# Supplementary material for: Achene Morphology and Anatomy of Clematis L. (Ranunculaceae) in Korea and Its Taxonomic Implications
Source: Plants (Basel). 2020 Sep 28;9(10):1279. doi: 10.3390/plants9101279 (PMC7650620; doi:10.3390/plants9101279)
Supplement: Supplementary file 1 [file plants-09-01279-s001.zip › supplementary/Tables.doc]

**Table 1. Name of taxa with voucher number and collection information.**

| **Taxon** | **Locality** | **Voucher No.** |
| --- | --- | --- |
| *C. apiifolia* DC. | Mt. Sinbul, Icheon-ri, Sangbuk-myeon, Ulju-gun, Ulsan, Korea | Sinbulsan-190911-001 |
| *C. brevicaudata* DC. | Unchi-ri, Sindong-eup, Jeongseon-gun, Gangwon-do, Korea | Unchiri-191007-001 |
| *C. trichotoma* Nakai | Mt. Sinbul, Icheon-ri, Sangbuk-myeon, Ulju-gun, Ulsan, Korea | Sinbulsan-190911-001 |
| *C. taeguensis* Y. Lee | Gyuram-ri, Jeongseon-eup, Jeongseon-gun, Gangwon-do, Korea | Gyuramri-190818-001 |
| *C. hexapetala* Pall. | Ho-ri, Palbong-myeon, Seosan-si, Chungcheongnam-do, Korea | Hori-190809-001 |
| *C. terniflora* DC. | Jukpo-ri, Dolsan-eup, Yeosu-si, Jeollanam-do, Korea | Dolsando-191004-002 |
| *C. terniflora* var. *mandshurica* (Rupr.) Ohwi | Namhansanseong Fortress, Sanseong-ri, Namhansanseong-myeon, Gwangju-si, Gyeonggi-do, Korea | Namhansanseong-190809-001 |
| *C. heracleifolia* DC. | Sihwa Lake, Munho-ri, Namyang-eup, Hwaseong-si, Gyeonggi-do, Korea | Sihwaho-190921-016 |
| *C. urticifolia* Nakai ex Kitag. | Mt. Gariwang, Sugam-ri, Bukpyeong-myeon, Jeongseon-gun, Gangwon-do, Korea | Gariwangsan-191007-001 |
| *C. takedana* Makino | Sihwa Lake, Munho-ri, Namyang-eup, Hwaseong-si, Gyeonggi-do, Korea | Sihwaho-190921-001 |
| *C. patens* C.Morren & Dence. | Mt. Johang, Samsong-ri, Cheongcheon-myeon, Goesan-gun, Chungcheongbuk-do, Korea | Johangsan-170831-049 |
| *C. brachyura* Maxim. | Seondol, Bangjeol-ri, Yeongwol-eup, Yeongwol-gun, Gangwon-do, Korea | Seondol-190719-001 |
| *C. serratifolia* Rehder | Gasong-ri, Dosan-myeon, Andong-si, Gyeongsangbuk-do, Korea | Gasongri-191007-001 |
| *C. fusca* var. *fusca* Turcz. | Mt. Cheongtae, Sapgyo-ri, Dunnae-myeon, Hoengseong-gun, Gangwon-do, Korea | Cheongtaesan-190819-001 |
| *C. fusca* var. *flabellata* (Nakai) J. S. Kim | Eundae-bong, Gohan-ri, Gohan-eup, Jeongseon-gun, Gangwon-do, Korea | Eundaebong-190818-001 |
| *C. fusca* var. *violacea* Maxim. | Mt. Baekhwa, Mawon-ri, Mungyeong-eup, Mungyeong-si, Gyeongsangbuk-do, Korea | Baekhwasan-150707-007 |
| *C. calcicola* J. S. Kim | Mt. Deokhang, Daei-ri, Singi-myeon, Samcheok-si, Gangwon-do, Korea | Deokhangsan-190818-001 |
| *C. koreana* Kom. | Mt. Hambaek, Gohan-ri, Gohan-eup, Jeongseon-gun, Gangwon-do, Korea | Hambaeksan-190818-001 |
| *C. ochotensis* (Pall.) Poiret | Mt. Gariwang, Sugam-ri, Bukpyeong-myeon, Jeongseon-gun, Gangwon-do, Korea | Gariwangsan-190819-007 |

***Table 2.*** *Morphological and anatomical features of achene of Clematis.*

| **Taxa** | **Shape** | **Color** | **Achene indument** | **Lateral wings** | **Style elongation** | **Style** | **Surface sculpture** | **Surface cells outline** | **Periclinal wall** | **Anticlinal wall** |
| --- | --- | --- | --- | --- | --- | --- | --- | --- | --- | --- |
| *C. apiifolia* | Narrow-elliptical | Brown | Completely hairy, hairs short on the achene body and long on the style | Narrow | Elongated | Plumose | Striate-rugose | Not differentiated | Concave with fine folds | Raised, folded |
| *C. brevicaudata* | Elliptic | Brown | Completely hairy, hairs short on the achene body and long on the style | Narrow | Strongly elongated | Plumose | Striate-rugose | Not differentiated | Concave with fine folds | Raised, folded |
| *C. trichotoma* | Obovate to fusiform | Black | Glabrous | Narrow | Strongly elongated | Plumose | Striate-rugose | Not differentiated | Concave with fine folds | Slightly raised |
| *C. taeguensis* | Elliptical to obovate | Light yellow | Sparsely hairy, fine hair on the body and long on the style | Wide | Strongly elongated | Plumose | Striate-colliculate | Elongated, rectangular | Convex with fine folds | Sunken, smooth |
| *C. hexapetala* | Obovate | Dark brown to black | Completely hairy | Medium | Elongated | Plumose | Striate-rugose | Elongated | Concave with fine folds | Raised, folded |
| *C. terniflora* | Elliptic to Obovate | Brown | Sparsely hairy, fine hair on the body and long on the style | Wide | Elongated | Plumose | Striate-reticulate | Rectangular, polygonal | Concave with fine folds | Raised, smooth |
| *C. terniflora* var. *mandshurica* | Elliptic | Light yellow | Sparsely hairy, fine hair on the body and long on the style | Wide | Elongated | Plumose | Striate-reticulate | Rectangular, polygonal | Concave with fine folds | Sunken, smooth |
| *C. heracleifolia* | Obovate | Dark brown | Completely hairy | Narrow | Strongly elongated | Plumose | Striate-rugose | Irregular | Concave with fine folds | Raised, folded |
| *C. urticifolia* | Elliptic to Obovate | Light greenish yellow | Completely hairy | Medium | Strongly elongated | Plumose | Striate-rugose | Irregular | Concave with fine folds | Raised, folded |
| *C. takedana* | Obovate | Dark brown to black | Completely hairy | Medium | Strongly elongated | Plumose | Striate-rugose | Irregular | Concave with fine folds | Raised, folded |
| *C. patens* | Obovate | Dark brown to black | Completely hairy, hairs short on the achene body and long on the style | Medium | Strongly elongated | Plumose | Striate-rugose | Irregular | Concave with fine folds | Raised, folded |
| *C. brachyura* | Obovate | Brown, black | Sparsely hairy | Wide | Not elongated | Glabrous | Rugose, Pustulate | Elongated | Concave pustulate | Raised, pustulate |
| *C. serratifolia* | Obovate | Medium brown | Sparsely hairy, fine hair on the body and long on the style | Narrow | Strongly elongated | Plumose | Reticulate | Elongated, rectangular | Concave with fine folds | raised, smooth |
| *C. fusca* var. *fusca* | Obovate | Dark brown to black | Completely hairy | Wide | Strongly elongated | Plumose | Striate-reticulate | Polygonal | Concave with fine folds | Raised smooth or fine folds |
| *C. fusca* var. *flabellata* | Elliptic to Obovate | Brown | Completely hairy | Medium | Strongly elongated | Plumose | Striate-reticulate | Polygonal | Concave with fine folds | Slightly raised, smooth |
| *C. fusca* var. *violacea* | Obovate | Black | Completely hairy | Medium | Strongly elongated | Plumose | Striate-reticulate | Polygonal | Concave with fine folds | Raised smooth or fine folds |
| *C. calcicola* | Narrow-elliptical | Dark brown | Completely hairy | Narrow | Strongly elongated | Plumose | Reticulate | Polygonal | Concave with fine folds | Raised smooth or fine folds |
| *C. koreana* | Obovate | Medium brown | Completely hairy | Narrow | Strongly elongated | Plumose | Reticulate | Elongated, polygonal | Concave with fine folds | Raised smooth or fine folds |
| *C. ochotensis* | Obovate | Brown | Sparsely hairy, fine hairs on the body and long on the style | Narrow | Strongly elongated | Plumose | Reticulate | Elongated, polygonal | Concave with fine folds | Raised smooth or fine folds |

**T**able 2. contd.

| **Taxa** | **Outline in cross section** | **Exocarp** | **Mesocarp** | **Endocarp** | **Seed coat** |
| --- | --- | --- | --- | --- | --- |
| *C. apiifolia* | Elliptic | Degenerating cells | Few layers of degenerating cells | Thin, single layer of lignified cells | Degenerated |
| *C. brevicaudata* | Elliptic | Cutinized single tanniniferous layer | 3-4 layers of parenchyma layer with wavy wall | Thick, single layer (rarely double layer) sclerid cells | Few layers of parenchyma cells |
| *C. trichotoma* | Elliptic | Cutinized single tanniniferous layer | 8-10 layers of thin walled parenchyma cells with wavy wall | Thin, single layer of lignified cells | 1-2 layers of degenerating cells |
| *C. taeguensis* | Dumbbell | Cutinized single layer parenchyma cells with thick outer wall | 4-6 layers of thin walled parenchyma cells with wavy wall | Thick, single layer of sclerid cells | 5-7 layers of parenchyma cells |
| *C. hexapetala* | Fusiform | Cutinized single tanniniferous layer | Few layers of degenerating cells | Thick, single layer of sclerid cells | Few layers of degenerating cells |
| *C. terniflora* | Dumbbell | Cutinized single layer parenchyma cells with thick outer wall | 2-4 layers of thin walled parenchyma cells with wavy wall | Single layer of sclerid cells | Few layers of degenerating cells |
| *C. terniflora* var. *mandshurica* | Dumbbell | Cutinized single layer parenchyma cells with thick outer wall | 4-6 layers of thin walled parenchyma cells with wavy wall | Thick, single layer of sclerid cells | Few layers of parenchyma cells |
| *C. heracleifolia* | Fusiform | Cutinized single layer parenchyma cells with thick outer wall | 4-6 layers of thin walled parenchyma cells with wavy wall | Thick, single layer of lignified palisade cells | Few layers of degenerating cells |
| *C. urticifolia* | Elliptic | Cutinized single layer parenchyma cells with thick outer wall | 4-6 layers of thin walled parenchyma cells with wavy wall | Single layer of sclerid cells | Few layers of degenerating cells |
| *C*. *takedana* | Elliptic | Cutinized single layer parenchyma cells with thick outer wall | 6-8 layers of thin walled parenchyma cells with wavy wall | Thin, single layer of lignified cells | 5-7 layers of parenchyma cells |
| *C. patens* | Fusiform | Cutinized single tanniniferous layer | Few layers of degenerating cells | Thick, single layer of lignified palisade cells | Few layers of parenchyma cells |
| *C. brachyura* | Narrowly fusiform | Cutinized single layer parenchyma cells with thick outer wall | 2-4 layers of thin walled parenchyma cells with wavy wall | Thick, single layer (rarely double layer) sclerid cells | 3-5 layers of parenchyma cells |
| *C. serratifolia* | Fusiform | Cutinized single layer parenchyma cells with thick outer wall | Few layers of degenerating cells | Thick, single layer of lignified palisade cells | Few layers of degenerating cells |
| *C. fusca* var. *fusca* | Fusiform | Cutinized single taniniferous layer | Few layers of degenerating cells | Single layer of sclerid cells | 5-7 layers of parenchyma cells |
| *C. fusca* var. *flabellata* | Fusiform | Cutinized single taniniferous layer | Few layers of degenerating cells | Thick, single layer (rarely double layer) sclerid cells | Few layers of degenerating cells |
| *C. fusca* var. *violacea* | Fusiform | Degenerated | Few layers of degenerating cells | Thick, single layer of lignified palisade cells | Few layers of degenerating cells |
| *C. calcicola* | Oval, elliptical | Cutinized single layer parenchyma cells with thick outer wall | 6-8 layers of thin walled parenchyma cells with wavy wall | Thick, single layer (rarely double layer) sclerid cells | Few layers of degenerating cells |
| *C. koreana* | Oval, elliptical | Cutinized single layer parenchyma cells with thick outer wall | 6-8 layers of thin walled parenchyma cells with wavy wall | Thick, single layer (rarely double layer) sclerid cells | 3-5 layers of parenchyma cells |
| *C. ochotensis* | Oval, elliptical | Cutinized single layer parenchyma cells with thick outer wall | 2-3 layers of thin walled parenchyma cells with wavy wall | Thin, single layer (rarely double layer) sclerid cells | Few layers of degenerating cells |

**Table 3.** Achene characters and their measurements in *Clematis* taxa (mean and standard deviation). L/W = length and width ratio, P/E = pericarp and endocarp ratio, S/B = style and achene body ratio, CSD1 = diameter parallel to cotyledon, CSD2 = diameter perpendicular to cotyledon, D1/D2 = CSD1 and CSD2 ratio.

| **Taxa** | **Length** | **Width** | **L/W** | **Pericarp** | **Endocarp** | **P/E** | **Style** | **S/B** | **CSD1** | **CSD2** | **CSD1/CSD2** |
| --- | --- | --- | --- | --- | --- | --- | --- | --- | --- | --- | --- |
| *C. apiifolia* | 3.95±0.4 | 1.32±0.2 | 3.01±0.21 | 63.51±15.58 | 6.32±1.06 | 10±1.36 | 9.55±1.29 | 2.44±0.38 | 1.66±0.04 | 1.08±0.07 | 1.54±0.08 |
| *C. brevicaudata* | 2.53±0.19 | 1.62±0.18 | 1.59±0.21 | 104.89±26.35 | 12.03±3 | 8.99±2.56 | 25.74±3.24 | 10.17±1.17 | 2.07±0.05 | 1.15±0.09 | 1.8±0.15 |
| *C. trichotoma* | 4±0.27 | 2.67±0.24 | 1.5±0.05 | 343.62±68.40 | 9.97±1.52 | 35.94±6.86 | 27.74±3.04 | 6.95±0.83 | 3.06±0.28 | 2.17±0.39 | 1.43±0.16 |
| *C. taeguensis* | 4.3±0.47 | 3.3±0.48 | 1.34±0.21 | 327.13±46.45 | 12.06±1.64 | 27.19±2.17 | 35.35±3.48 | 8.25±1.2 | 3.28±0.08 | 1.15±0.1 | 2.87±0.24 |
| *C. hexapetala* | 4.73±0.4 | 2.88±0.2 | 1.64±0.04 | 56.52±11.77 | 13.47±1.93 | 4.17±0.35 | 15.01±2.33 | 3.21±0.63 | 2.93±0.21 | 1.38±0.19 | 2.13±0.14 |
| *C. terniflora* | 5.44±0.43 | 3.12±0.3 | 1.75±0.06 | 94.85±29.49 | 11.84±2.05 | 7.83±1.24 | 25.97±4.08 | 4.76±0.74 | 3.44±0.3 | 1.33±0.14 | 2.6±0.14 |
| *C. terniflora* var. *mandshurica* | 6.75±0.39 | 3.5±0.4 | 1.94±0.13 | 84.89±16.41 | 9.84±1.43 | 8.72±1.65 | 31.66±3.24 | 4.71±0.55 | 3.73±0.21 | 1.47±0.12 | 2.55±0.21 |
| *C. heracleifolia* | 3.21±0.36 | 2.01±0.25 | 1.6±0.0.08 | 136.06±25.36 | 25.61±4.41 | 5.3±0.17 | 17.07±2.55 | 5.4±1.21 | 3.01±0.48 | 1.42±0.12 | 2.21±0.18 |
| *C. urticifolia* | 3.83±0.25 | 2.9±0.27 | 1.33±0.09 | 285.8±41.04 | 16.35±3.12 | 17.24±4.21 | 26.55±3.8 | 6.97±1.25 | 2.89±0.37 | 1.49±0.09 | 1.94±0.33 |
| *C. takedana* | 2.88±0.38 | 1.86±0.22 | 1.56±0.21 | 232.31±52.36 | 9.94±2.27 | 23.92±5.1 | 26.23±3.7 | 9.3±1.64 | 2.08±0.08 | 1.27±0.16 | 1.66±0.26 |
| *C. patens* | 4.04±0.3 | 3.68±0.51 | 1.12±0.09 | 183.29±18.69 | 57.69±7.41 | 3.33±0.83 | 30.21±5.17 | 7.51±1.41 | 4.95±0.2 | 2.45±0.13 | 2.03±0.18 |
| *C. brachyura* | 9.12±0.88 | 7.3±0.46 | 1.25±0.10 | 142.19±28.08 | 22.44±3.39 | 6.37±1.04 | 2.99±0.55 | 0.33±0.07 | 7.1±0.14 | 1.08±0.11 | 6.65±0.6 |
| *C. serratifolia* | 2.86±0.17 | 1.42±0.13 | 2.02±0.11 | 41.44±6.93 | 12.83±2.09 | 3.23±0.14 | 27.16±4.52 | 9.51±1.62 | 1.61±0.04 | 1.04±0.1 | 1.6±0.12 |
| *C. fusca* var. *fusca* | 4.64±0.41 | 2.99±0.28 | 1.52±0.07 | 89.63±25.9 | 13.29±1.93 | 6.9±2.26 | 28.61±6.65 | 6.25±1.71 | 3.58±0.17 | 1.21±0.08 | 2.96±0.2 |
| *C. fusca* var. *flabellata* | 3.77±0.3 | 2.49±0.23 | 1.14±0.06 | 77.92±25.31 | 12.51±2.71 | 6.3±0.95 | 35.71±4.9 | 9.55±1.72 | 2.86±0.11 | 1.65±0.08 | 1.73±0.02 |
| *C. fusca* var. *violacea* | 6.1±0.7 | 5.34±0.51 | 1.18±0.38 | 70.3±12.45 | 34.1±3.54 | 2.05±0.16 | 44.74±5.81 | 7.37±0.9 | 5.16±0.15 | 1.92±0.32 | 2.73±0.34 |
| *C. calcicola* | 4.44±0.48 | 2.07±0.25 | 2.18±0.38 | 222.95±25.82 | 24.27±4.22 | 9.35±2.25 | 28.48±4.29 | 6.48±1.23 | 2.86±0.25 | 1.85±0.3 | 1.59±0.33 |
| *C. koreana* | 4.77±0.35 | 2.55±0.36 | 1.88±0.12 | 111.82±26.78 | 27.66±3.51 | 4.11±0.46 | 31.85±4.95 | 6.7±1.14 | 2.36±0.07 | 1.66±0.08 | 1.42±0.04 |
| *C. ochotensis* | 3.76±0.25 | 1.64±0.26 | 2.35±0.39 | 111.83±16.44 | 12.57±1.5 | 8.96±1.42 | 28.56±2.45 | 7.64±0.89 | 1.81±0.19 | 1.29±0.15 | 1.42±0.3 |
| **ANOVA** | **F=206.22, *p*<0.001** | **F=298.9,**  ***p*<0.001** | **F=101.98, *p*<0.001** | **F=121.01,**  ***p*<0.001** | **F=182.24, *p*<0.001** | **F=190.03, *p*<0.001** | **F=83.7, *p*<0.001** | **F=72.68, *p*<0.001** | **F=210.17, *p*<0.001** | **F=26.89, *p*<0.001** | **F=114.58, *p*<0.001** |
